# Chronic Self-Myofascial Release in Road Cyclists: Effects on Cardiorespiratory Capacity, Metabolism, and Mechanical Power

**DOI:** 10.3390/sports14020082

**Published:** 2026-02-13

**Authors:** Doris Posch, Markus Antretter, Martin Burtscher, Martin Faulhaber

**Affiliations:** Department of Sport Science, University of Innsbruck, Fürstenweg 185, A-6020 Innsbruck, Austria; markus.antretter@gmx.at (M.A.); martin.burtscher@uibk.ac.at (M.B.); martin.faulhaber@uibk.ac.at (M.F.)

**Keywords:** foam rolling, massage, endurance, mechanical performance, lactate kinetics, recreational cyclists, cycling training, fascia, recovery

## Abstract

**Background**: Foam rolling is a popular self-myofascial release (SMR) technique, yet empirical evidence regarding its long-term impact on cycling endurance remains inconclusive. This study investigated the effects of chronic SMR on cardiorespiratory capacity, metabolic kinetics, and mechanical performance in road cyclists. **Methods**: We conducted a six-month randomized controlled trial (RCT) with 32 male recreational cyclists. Both an intervention group (IG) and a control group (CG) followed a standardized training protocol. The IG additionally applied a Blackroll^®^ foam roller immediately after cycling training sessions. Outcomes included maximum oxygen uptake (VO_2_max), submaximal heart rate, lactate slope, and relative mechanical power (W/kg) at aerobic and anaerobic thresholds. Data were analyzed using linear mixed-effects models (LMM), with age included as a fixed-effect covariate to control for baseline imbalances between groups. Effect sizes were determined via marginal and conditional R^2^. Additionally, model robustness was verified through Shapiro–Wilk tests and Q–Q plots of conditional residuals. **Results**: No significant effects were observed for VO_2_max or submaximal heart rate. In contrast the IG demonstrated significant improvements in metabolic kinetics, evidenced by a reduced lactate slope (*p* = 0.004). Furthermore, foam rolling yielded a statistically significant positive effect on relative mechanical performance at both the aerobic (*p* = 0.031) and anaerobic (*p* = 0.007) lactate thresholds. Sensitivity analyses confirmed that these effects were independent of the age difference between groups. **Conclusions**: Foam rolling did not enhance all endurance-related variables but showed positive effects on metabolic kinetics and mechanical performance. While it did not shift systemic cardiorespiratory limits, SMR appeared to optimize performance through improved metabolic economy and mechanical efficiency, suggesting it is a valuable supplemental tool for recovery and long-term performance maintenance in cycling.

## 1. Introduction

The use of foam rollers for self-myofascial release (SMR) has become a popular technique among athletes and physiotherapists to reduce post-training muscular pain and improve athletic performance [1]. Foam rollers are self-massage devices by which the targeted fascia is rolled and compressed, utilizing the athlete’s own body weight to apply pressure to the soft tissues. This process stretches the tissue and creates friction, similar to a conventional massage [2]. The term *fascia* refers to the fibrous connective tissue that penetrates and surrounds muscles, organs, bones, nerves, and blood vessels in the form of a complex, three-dimensional network [3]. Current evidence suggests that the musculoskeletal system should be viewed as an integrated ‘myofascial unit’, where the fascia plays a dynamic role in force transmission and intramuscular coordination [4]. Recent research has further elucidated that fascia is not merely a passive wrapping tissue but a highly specialized, sensory organ with significant roles in force transmission and proprioception [5,6]. The fascial system is richly innervated with mechanoreceptors and free nerve endings, making it a key player in nociception and autonomic regulation [7].

This network is a dynamic, contractile tissue rather than a passive material. Due to trauma, inflammation, or immobility, the fascia can lose flexibility and become restricted, resulting in myofascial imbalances, pain, or joint dysfunction [8]. Furthermore, the role of the extracellular matrix and the viscosity of hyaluronic acid between fascial layers have been identified as crucial factors for inter-muscular gliding and movement efficiency [4,9]. Consequently, various theories attempt to explain the benefits of myofascial release, which can be subdivided into neurological, mechanical, physiological, and psychophysiological explanations [2]. According to mechanical models, SMR may lead to a reduction in tissue adhesion, thixotropic effects, or altered tissue stiffness [10,11,12]. The thixotropic property of the fascial ground substance describes its ability to become more fluid when subjected to mechanical stress, which is discussed as a potential factor in reducing internal resistance during repetitive movements like cycling [13].

Physiological explanations suggest that foam rolling increases mobility, promotes blood flow, and improves vascular endothelial function. It is also postulated that SMR leads to increased parasympathetic activity, which may reduce inflammation and fascial tension, thereby aiding recovery [2,11]. These assumptions regarding enhanced microcirculation are supported by ultrasonographic research demonstrating significant increases in arterial tissue perfusion and blood flow velocity immediately following SMR [14,15]. Such an increase in volume flow theoretically expands the capacity for lactate transport from the working musculature into the vascular system. A very recent study by Alansari et al. further demonstrated that SMR significantly enhances metabolic recovery by accelerating lactate reduction and normalizing muscle temperature, monitored via thermal imaging, more effectively than passive recovery strategies [16].

Beyond localized effects, it is hypothesized that SMR exerts systemic influence through altered interstitial pressure and neurophysiological signaling. The compression generated by a roller is suggested to enhance local microcirculation by reducing myofascial resistance and improving the fluidity of the ground substance [13,17]. In this context, studies provide empirical evidence that SMR can significantly accelerate lactate clearance following high-intensity exercise, indicating an optimized metabolic recovery rate [18,19]. Additionally, evidence shows that even a single bout of SMR confers cardiovascular benefits, affecting peripheral and central blood pressure as well as arterial stiffness [20]. This improved blood flow is hypothesized to optimize lactate kinetics by facilitating the transport of metabolic byproducts from the interstitial space back into the vascular system.

Furthermore, the integration of SMR into a training regimen may influence cardiorespiratory efficiency through the modulation of the autonomic nervous system. By stimulating mechanoreceptors within the fascial network, specifically Ruffini and Pacini corpuscles, SMR could potentially shift the athlete’s state toward parasympathetic dominance [21,22]. This reduction in systemic sympathetic drive may manifest as improved heart rate recovery and lower submaximal oxygen cost. Therefore, the measurement of maximum oxygen uptake (VO_2_max) and cardiorespiratory efficiency in this study evaluates whether these tissue-level interventions translate into a more efficient systemic “input” during sustained aerobic work.

Neurological theories emphasize that foam rolling may reinforce analgesic effects and muscle recovery by mediating pain-modulatory systems, including diffuse noxious inhibitory control and mechanoreceptor sensitivity [2,23,24]. Finally, psychophysiological models argue that the positive impact may be explained by increased plasma endorphins or decreased arousal levels [25]. These mechanisms are likely interlinked; for instance, a friction-induced increase in tissue temperature leads to a thixotropic response, while improved oxygenation reduces the likelihood of trigger point formation [1]. In cycling, the impact of SMR on mechanical endurance must be viewed through the lens of movement economy. Chronic fascial restrictions can lead to suboptimal recruitment patterns, whereas maintaining fascial fluidity may preserve the efficiency of the pedal stroke [21]. Additionally, SMR can increase the electromyographic fatigue threshold, suggesting a more efficient muscle activation pattern and a delay in the recruitment of higher-order motor units [10]. This study explores the nexus between these interventions and the lactate threshold (LT) as the ultimate integration of cardiorespiratory capacity, metabolic efficiency, and mechanical economy.

Despite the popularity of these models, empirical evidence remains inconclusive, particularly regarding longitudinal effects in cycling [26]. Some research concluded that SMR does not improve muscle performance in a randomized cross-over design [27]. In contrast, other trials found that acute SMR alleviates pain and enhances motor performance and flexibility in cyclists [28,29]. Similar conflicts exist in other sports; while effects on muscle function remain unclear, a growing body of literature suggests that SMR alleviates muscle soreness and improves endurance recovery [2]. This lack of clarity stems from the infancy of fascial research, a shortage of high-quality longitudinal studies, and the diversity of research protocols applied across studies [2,11,26]. Such conflicting results can also be observed in studies focusing on sports other than cycling. A systematic meta-analysis of the scholarly literature conducted by Wiewelhove et al. points out that, on the one hand, the effects of foam rolling on muscle function remain unclear due to inconclusive empirical results, while, on the other hand, a growing body of literature suggests that self-myofascial release using foam rollers or roller massage sticks alleviates muscle soreness. As the authors conclude, post-rolling improves endurance and strength performance and reduces perceived muscle pain [2].

The present study investigated the effects of a six-month foam rolling intervention using the Blackroll^®^ (BLACKROLL AG, Bottighofen, Switzerland) on endurance indicators in cyclists, bridging the gap between localized tissue treatment and global athletic output. We developed a comprehensive framework to test the primary assumption that SMR improves endurance-related variables. First, we focus on cardiorespiratory capacity, which reflects the systemic “input” of the athlete via maximum oxygen uptake (H1) and cardiorespiratory efficiency via submaximal heart rate at fixed lactate thresholds (H2). Second, we address metabolic kinetics (H3) by analyzing the lactate curve progression between 100 and 250 watts. Finally, we investigate mechanical performance (H4) at the aerobic and anaerobic lactate thresholds. This parameter represents the ultimate integration of an athlete’s cardiorespiratory capacity, metabolic efficiency, and mechanical economy. By analyzing these four pillars, this study seeks to determine whether the chronic SMR provides a measurable ergogenic advantage.

## 2. Materials and Methods

### 2.1. Study Design

The study design consisted of a randomized controlled trial (RCT) over a six-month period, which is widely regarded as the gold standard for investigating the efficacy or effectiveness of a treatment or intervention [30,31]. Recruitment took place between mid-March and mid-September (weeks 12–38). First, to ensure the reliability and objectivity of the measurements, a preliminary trial with twelve recreational cyclists was conducted, organized by our research team, to standardize all experimental procedures including the precise control and standardization of the seat position on the road bikes. These internal pre-tests served as a crucial quality control measure to ensure consistent biomechanical conditions for all participants throughout the trial.

Two weeks prior to data collection (calendar weeks 38–39), a systematic bike-fitting was conducted for all participants to optimize cycling position regarding performance, comfort, and injury prevention. Since saddle geometry significantly governs pressure distribution [32], muscle recruitment, and intermuscular dynamics [33], strict standardization was essential to eliminate positioning bias [34]. The adjustment process followed a two-stage protocol. In the first, static stage, four anatomical reference points—the greater trochanter, lateral epicondyle, lateral malleolus, and the fifth metatarsal head—were marked. The static foundation consisted of a horizontally leveled saddle and a modern cleat positioning centered between the first and fifth metatarsal heads [35,36], deviating from the traditional positioning according to Silberman et al. [37]. Saddle height was set to 107–109% of inseam length [38], while saddle setback was standardized using the KOPS method (Knee Over Pedal Spindle), ensuring a plumb line from the patella passed through the pedal spindle at the 3 o’clock crank position [39]. Target parameters included a trunk inclination of 40°–45° to the horizontal [40], a knee flexion angle of 35° at the bottom dead center [41], and a plantar flexion between 15° and 30° [36].

In the second, dynamic stage, the position was validated using Kinovea analysis software (Version 0.9.3) [42]. Participants were tested on their own bicycles, with the back wheel mounted on a magnetic resistance indoor trainer. Following a 5 min warm-up at 100 W, angular stability was verified over a 60 s measurement period at 150 W and a cadence of 80 RPM. Due to the high experience level of the ambitious recreational athletes, only minor adaptations were generally required. This dual approach of static reference measurement and dynamic tracking ensured that power output and VO_2_max reflected the participants’ actual physiological capacity, free from biomechanical confounding factors. This preparatory phase, including final recruitment, bike fitting and training instructions, was finalized for all subjects by the end of September (calendar weeks 37–40).

**Figure 1:**
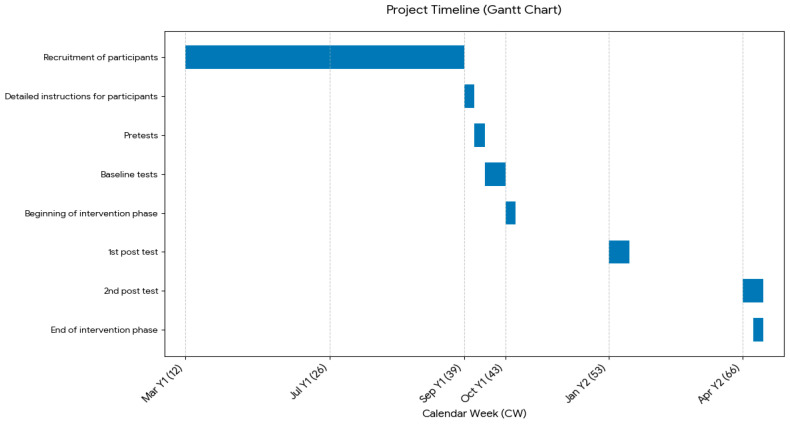
Timeline of the project, with blue bars marking the periods of specific project stages (own illustration).

Baseline assessments were conducted in early October (weeks 41–42), immediately prior to the intervention launch in mid-October (week 43). The study design included two follow-up assessments. The main post-test, which was the second follow-up assessment, was conducted six months after the intervention began, in April (week 13 for the intervention group and weeks 13–14 for the control group). An interim post-test was also scheduled halfway through the intervention, three months after the trial began. However, the interim assessment at three months was intentionally restricted to body composition and biomedical indicators, such as BMI, weight distribution as well as leg circumference. This decision was primarily based on the hypothesized time course of fascial adaptation; while SMR is known to induce immediate thixotropic responses in the fascial ground substance [13], the structural remodeling of the complex, three-dimensional network of collagen fibers is considered a chronic process [3]. Evidence suggests that long-term fascia training (spanning six months to two years) is required to substantially improve movement patterns and coordination, resulting in more efficient muscle function and improved overall performance [21]. By contrast, variables related to performance and endurance, such as oxygen uptake, watts per kg or heart rate at different lactate thresholds were measured exclusively at the beginning of the trial (baseline) and during the final post-test six months later. To evaluate whether long-term myofascial release leads to these systemic changes in cardiorespiratory efficiency and metabolic kinetics, a six-month observation period was deemed more appropriate to capture these structural adaptations. Additionally, this approach minimized the physiological burden of repeated maximal exercise testing and ensured high participant compliance throughout the study. Figure 1 provides an overview of the project timeline.

In our study 32 male recreational road race cyclists—16 participants were randomly assigned to an intervention group, in which they applied a Blackroll^®^ (BLACKROLL AG, Bottighofen, Switzerland) foam roller immediately after two tightly controlled cycling training sessions per week, and 16 to a control group, who followed the same cycling training protocol but without using the Blackroll^®^ as a post-rolling myofascial release technique or any other type of massage. The intervention group was instructed to use the Blackroll^®^ on different body parts according to a tightly controlled protocol.

### 2.2. Recruitment Protocol and Selection Criteria

As first step the statistical power calculations were performed using the free software G*Power (Version 3.1.9.7) (Heinrich-Heine-University, Düsseldorf, Germany) [43]. The target parameters were set to an anticipated medium effect size of Cohen’s *d* = 0.50, a two-tailed significance level of *α* = 0.05, and a desired statistical power of 1 − *β* = 0.80. The a priori power analysis determined that a required total sample size (*N_req_*) of approximately 128 participants would have been necessary to achieve the target power under the assumption of an infinite population. The total population was strictly limited based on predefined inclusion criteria. This total population of *N_Pop_* = 46 individuals was identified beforehand through a questionnaire distributed to all relevant associations in Tyrol, Austria.

The selection of participants was guided by predefined inclusion criteria. We used a non-probabilistic sampling procedure in the form of consecutive sampling. To qualify for the study, athletes needed to be recreational cyclists aged between 25 and 59 with consistent training for at least three years. A primary requirement was year-round cycling training with a frequency of 3–4 sessions per week, including mandatory indoor training during the winter months to ensure no seasonal interruptions. Additionally, participants were required to provide a verified training documentation (digital or diary-based) covering at least the 12 months prior to the start of the study. Furthermore, they were required to maintain an average weekly training load of 8–10 h, which corresponds to a self-reported mileage of approximately 250–300 km per week during the season and a total annual distance of approximately 12,000–15,000 km. Participants had to prove that no interruptions exceeding six weeks occurred in the past three years. Other criteria included experience in structured, power-based training, a spiroergometry test completed within the past two years, and at least six months of experience using bilateral power measuring pedals. Participants also had to be naïve to myofascial massage techniques with a foam roller prior to the trial.

Exclusion rules were established to enhance participant safety and ensure sample homogeneity. Cyclists were not eligible if they held a professional cycling license or if they had any of the following conditions: osteoporosis, thrombosis, fibromyalgia, disk injury, soft tissue rheumatism, uncontrolled hypertension, or joint implants in the hip or knee. To address the risk of bias through self-reporting, the absence of these conditions and the suitability for high-intensity exertion had to be confirmed via a mandatory medical clearance (Health Certificate) issued by a sports physician within two months prior to the start of the study. Participants could also be excluded during the trial if they were diagnosed with a new medical condition, became ill, withdrew their consent, did not comply with the protocol, or experienced intervention-related complications such as adverse reactions.

The study focused on adult male recreational cyclists, who were recruited in collaboration with cycling clubs across Tyrol, Austria. To reach the target population, an invitation email was circulated to all 35 officially registered clubs in the region, with enrollment taking place between mid-March and mid-September. Although female athletes were initially considered for inclusion, the limited number of respondents (only three women) led to their exclusion from the sample. A total of 36 men enrolled and were randomly divided into two equal groups of 18 participants each (intervention vs. control). Two participants of each group were excluded from the analyses because they dropped out and missed the post-test.

This left a final sample of 32 participants, 16 participants each group, representing a substantial ≈ 69.6% of the total population. The baseline characteristics of the final sample are summarized in Table 1.

To account for this high sampling fraction, a post hoc power analysis was conducted incorporating the Finite Population Correction (FPC). This correction is necessary because the standard error of a sample decreases when it represents a significant portion of a finite population [44]. By applying the FPC formula, which adjusts the variance relative to the population size (*N_Pop_* = 46), the analysis revealed that the actual statistical power to detect a medium effect of d = 0.50 was 72.4%. While this explains the difference from the initial a priori estimate of 128 (which assumed an infinite population), it should be noted that although this value falls short of the conventional 80% threshold, the high power is deemed statistically justifiable given the exceptional proportion of the population sampled. Nevertheless, the study is formally underpowered, and the risk of a Type II error (*β* = 27.6%) should be considered. Conversely, the study possessed the statistical strength to detect effects of only *d* ≈ 0.55 or larger with 80% power. While the achieved power of 72.4% was statistically justified using the Finite Population Correction (FPC), we primarily classify this investigation as an exploratory pilot study due to the shortfall from the original sample size planning (*n* = 128). This applies particularly to the evaluation of central cardiorespiratory parameters. Therefore, while the achieved power of 72.4% is high, the absence of statistically significant results should be interpreted cautiously as a potential consequence of a Type II error rather than conclusive evidence for the absence of a true medium effect.

**Figure 2:**
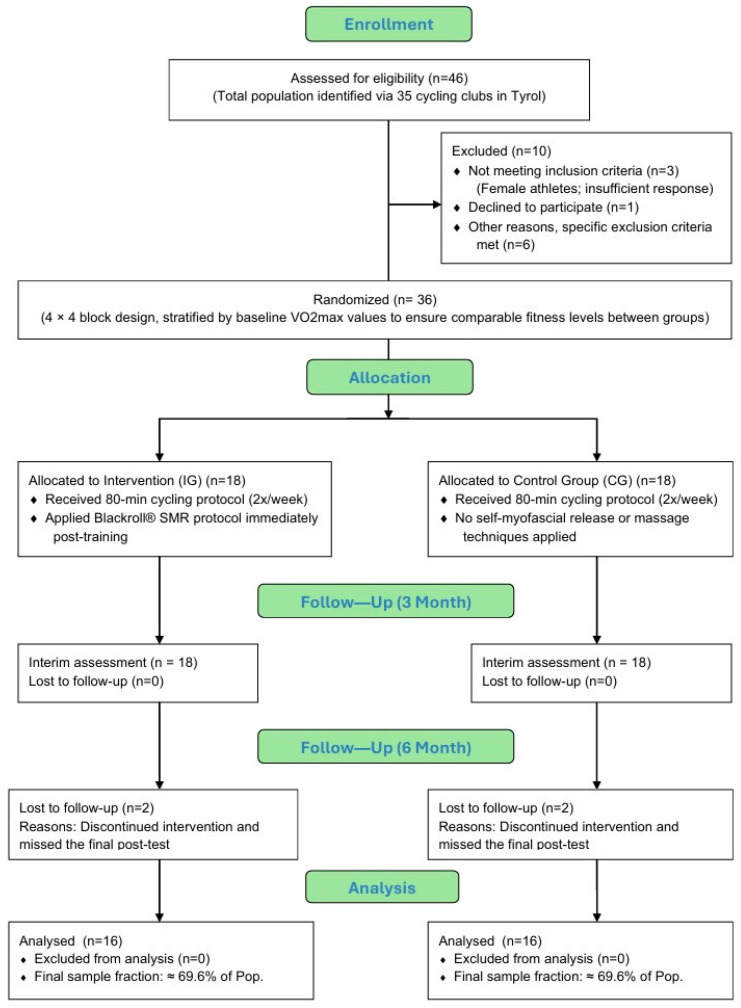
CONSORT flow diagram of participant recruitment and retention rates (own illustration).

The assignment to each group was carried out using a randomization process with a 4 × 4 block design, in which baseline VO_2_max values were used to stratify participants. In doing so, athletes were ranked according to their maximum oxygen intake in descending order, grouped into sets of four and then distributed evenly across the two groups. This procedure helped maintain a comparable baseline fitness level between the two groups. The following diagram illustrates the flow of participants through the study, beginning with the initial assessment of 46 individuals from 35 cycling clubs in Tyrol, Austria. It details the transition from enrollment to the randomization of 36 participants. The chart further tracks the 6-month follow-up period and documents the retention rates and the final sample fraction, which represents approximately 69.6% of the population, leading to the final analysis of the sample (Figure 2).

### 2.3. Testing Procedures

All testing sessions were conducted at the laboratories of the Faculty of Psychology and Sport Science at the Leopold-Franzens University Innsbruck, Innsbruck, Austria. To ensure high internal validity and control for circadian influences, all assessments were performed during the morning hours, with each participant’s follow-up tests scheduled at the same time of day as their baseline measurement. The laboratory environment was strictly controlled at a constant room temperature between 18 °C and 20 °C. Furthermore, all procedures followed a strictly standardized block sequence rather than a randomized order to protect the integrity of resting physiological markers. Each session began with anthropometric and localized biomedical measurements in a rested state, followed by performance diagnostics, and concluded with a health status evaluation.

All anthropometric assessments (height, body mass, circumference of thigh and lower leg, right/left weight distribution) were performed by the same trained investigators at the beginning of each testing session. Body height was measured only while baseline test to the nearest of 0.1 cm by using a portable stadiometer Seca 220 (Seca, Hamburg, Germany). Body mass was determined to the nearest of 0.1 kg using a portable scale TBF-531 (Tanita, Sindelfingen, Germany). Thigh and calf circumferences were measured bilaterally using a flexible, inelastic tape measure while the subjects lay on a treatment table with their legs bent at 90°. Thigh measurements were taken at the mid-thigh (i.e., at 50% of the distance between the greater trochanter and the lateral epicondyle of the femur). The circumference of the calf muscles was measured at the widest point of the lower leg. Right/left weight distribution was measured using two identical digital scales (Soemer, Lennestadt, Germany) while standing barefoot, hip-width apart, looking straight ahead.

Performance testing was carried out indoors on the athletes’ own racing bicycles, which were mounted on a CYCLUS 2 ergometry system (RBM, Leipzig, Germany). As established in the preliminary trials, the pre-calibrated seat geometry was maintained to ensure biomechanical consistency. Diagnostic testing via spiroergometry was conducted using an incremental test, beginning at 100 watts and increasing by 50 watts every three minutes. Notably, while the baseline and the 6-month final test included this performance diagnostic, the first post-test (3-month interim assessment) followed the exact same block sequence and standardized conditions but excluded the spiroergometry. The incremental protocol (3 min stages) followed the recommendations of Heck et al. and Bentley et al. [45,46]. This duration ensures the attainment of a physiological steady state and prevents a time-lag in lactate response, which is essential to avoid overestimating metabolic thresholds. Furthermore, the 50–watt increments allow the test to be completed within a valid timeframe of 15–25 min, preventing premature fatigue [47,48]. The test had to be completed at a cadence between 90 and 100 RPM. The test was terminated when the cadence could no longer be maintained and fell below 80 RPM. Cardio-respiratory measurements were conducted using an open spirometric system (Meta Lyzer 3B, CORTEX^®^, Biophysik, Leipzig, Germany). Blood lactate concentrations were analyzed from capillary blood samples taken from the earlobe (Super GL Ambulance, Dr. Müller Gerätebau, Freital, Germany). Lactate measurement was carried out at rest before the start of the test, at the end of each stage and 3 min after the test termination. Maximum blood lactate concentration (BLAmax) was defined as the value of the last sample 3 min after the end of the test. Blood lactate values were transferred to automated software winlactat (Mesics, Münster, Germany) for analysis of the lactate levels and lactate thresholds. To identify aerobic and anaerobic thresholds at 2 and 4 mmol/L, we applied the Mader method [49,50].

VO_2_max was defined as the average oxygen uptake over the highest 30 s interval. Additionally, we used the Garmin^®^ Vector^TM^ 2 power meter pedals (GARMIN GmbH, Würzburg, Germany) were used in conjunction with a Garmin^®^ Edge^®^ 1000 bike computer on the participants’ own bicycles. This setup recorded bilateral power output and heart rate throughout all tests and training sessions. Findings from Nimmerichter et al. suggest that the Garmin Vector 2 represents a valid alternative for training purposes. The study confirms that the Garmin Vector 2 pedals show a high correlation with gold-standard devices, such as the SRM system, particularly during steady-state submaximal efforts and overall performance testing [51].

To monitor and control training intensity, a dual-method approach was implemented. Subjective intensity was recorded using the 10-point Borg scale (RPE scale), ranging from 0 (‘nothing at all’) to 10 (‘extremely strong’) [52]. Simultaneously, objective physiological load was quantified via a modified 4-zone TRIMP model. This specific model subdivides the intensity range between the aerobic and anaerobic thresholds into two sub-zones (2a and 2b) with weighting factors of 2 and 3, respectively. This approach was chosen to accurately reflect the metabolic demands within the transition zone and to ensure a granular assessment of the training stimulus [53]. In regard to the intensity of the intervention with the Blackroll^®^ the Numeric Rating Scale (NRS) was used. The following Table 2 outlines the standardized assessment protocol, including the specific parameters measured at each timepoint and the chronological sequence of the testing blocks.

### 2.4. Training Protocol

The cycling training followed a structured and standardized plan for recreational cyclists, designed by the author based on extensive expertise as a state-certified trainer and a qualified educator in movement and sport. The program integrated years of experience as a successful national and international cyclist, ensuring it met the high-quality standards of the Austrian Cycling Federation and the Austrian Federal Academies for Sport (BSPA’s). Therefore, the basic training principles are to first ensure consistency, then increase the training load during the training process, balance the intensity distribution during the week, both for the mesocycle and the season, and apply the basic principles of periodization and tapering [54]. Methodologically, the training was specifically tailored for performance enhancement during the first preparatory period (PP I), grounded in the principles of periodization and load management established by Friel and the power-based training concepts of Allen and Coggan [55,56]. Furthermore, the intensity distribution was informed by the scientific findings of training models as described by Seiler as well as Laursen and Jenkins [57,58].

Both groups followed a heart rate-controlled cycling program that was strictly supervised throughout the study. The plan was organized according to a block-periodization model, consisting of six consecutive training cycles of four weeks each. At the end of every block, the workload was adjusted upward by 5%, based on each participant’s baseline performance level at the 2 mmol/L lactate threshold. Throughout the trial strength endurance intervals (KA intervals) progressively increased in intensity, starting in the lower GA2 zone and ending in the upper GA2 range after six months.

Based on our training protocol, participants were required to train four times a week to reach a total weekly training volume of 8–10 h, which served as the standardized baseline level. This volume was split into two primary study-specific sessions lasting 80 min each (focused on GA1/GA2 intervals), supplemented by two additional individual low-intensity training (LIT) sessions, performed as continuous exercise within the GA1 zone. To ensure variety and adherence, one of these GA1 sessions was performed on the bicycle, while the second was performed as an alternative outdoor endurance activity (e.g., brisk walking, ski touring), also strictly within the GA1 heart rate range. All study-related cycling sessions were performed individually at the participants’ homes to ensure high ecological validity and integration into their daily routines. For these home-based sessions, participants used their own racing bicycles mounted on a CYCLUS 2 ergometry system with a fixed rear wheel. Specifically, the training prescribed for the study was performed within the GA1 and GA1/GA2 heart rate zones, precisely corresponding to lactate values between 2 and 4 mmol/L. This targeted the aerobic–anaerobic transition zone to ensure a standardized metabolic stimulus across all participants. The training intensity was prescribed individually based on heart rate zones, which were determined via spiroergometry and lactate measurements while the baseline test with lactate-based thresholds at 2 and 4 mmol/L using the Mader method.

Both groups were required to train consistently within the basic endurance zones (GA1 and GA2) at a cadence of 90–100 revolutions per minute (RPM). Each cycling session began with a 10 min warm-up in the lower GA1 zone, followed by multiple strength endurance intervals lasting three to five minutes, which were conducted in GA2 at a reduced cadence of 60–70 RPM. Recovery phases were matched to work durations (1:1 ratio) and performed in GA1 at 90–100 RPM.

To ensure strict adherence to the prescribed training intensity and volume in the home-based and outdoor settings, all sessions were digitally monitored via the Garmin Connect™ online platform. Each participant’s data, including heart rate, power output (for cycling), and cadence, were uploaded and reviewed weekly by the lead investigator. Compliance was verified by comparing the recorded time-in-zone against the individual training plan. A high level of adherence (>96% of sessions) was documented for all participants included in the study. Participants were required to keep a detailed training diary documenting all endurance-related activities performed outside the supervised sessions. In addition to this, they had to keep a food diary during the intervention period.

### 2.5. Intervention Protocol

Participants in the intervention group completed a structured program of self-myofascial release immediately after each training session, applying the Blackroll^®^ foam roller to the lower and upper limbs as well as the thoracolumbar fascia. The control group, by contrast, followed the identical training protocol, but did not use the foam roller or any other comparable self-massage methods. To preserve the validity of the study design, athletes in both groups were explicitly instructed to avoid additional manual therapies or self-applied fascial treatments outside the prescribed protocol.

Adherence to the SMR protocol was high throughout the six-month intervention period. Participants were required to document each completed session in a digital training log. On average, the intervention group completed 92.4% (*SD* = 3.8%) of the prescribed SMR sessions. To ensure protocol integrity, these digital logs were reviewed monthly by the research team, and any deviations were addressed immediately with the participants. This high compliance rate can likely be attributed to the provided video-guided instructions, which facilitated the integration of the routine into the participants’ daily recovery phase.

The Blackroll^®^ is a foam roller that is frequently used in self-myofascial release to enhance tissue elasticity and optimize fascial function. We used the “Standard 30 cm” model as it is, according to the manufacturer, particularly well-suited for beginners and for use after physical exertion [59]. Prior to the intervention, participants received comprehensive instructions on the proper use of the foam roller for different exercises. Once the trial began, they were required to follow a standardized routine consisting of twelve exercises recommended by the manufacturer. These targeted specific muscles and fascial regions, including the plantar fascia, calf muscles, tibialis anterior, quadriceps, hamstrings, adductors, iliotibial tract, psoas, gluteal group and both lower and upper sections of the back [60,61].

The foam roller exercises were performed on a gym mat to ensure sufficient stability and space. Participants were instructed to roll very slowly (3 cm per minute) and in a controlled manner to promote recovery and relaxation, the rolling direction was from distal to proximal. While no external metronome was used, the participants were provided with a comprehensive video tutorial that offered a 1:1 real-time demonstration of the correct rolling speed, ensuring high consistency and standardization across the group. Specifically, they were required to pause for several seconds on particularly tense or painful points (trigger points) to apply targeted pressure. On these points, participants performed ten small rolling movements of about three to five centimeters until the tension eased. For specific body regions, such as the tibialis anterior, athletes were specially instructed to focus on avoiding rolling over the bony prominences. Instead, they were told to slightly internally rotate the lower leg in order to direct the pressure specifically onto the muscle (lateral to the shin bone) [1].

Each exercise consisted of 20 repetitions across the target muscle, followed by a 30 s pause before the sequence was repeated a second time. The pressure exerted should be chosen to produce a tolerable or “pleasant” pain rather than excessive, sharp discomfort. Participants could self-regulate the pressure by supporting themselves with their arms or the non-active leg. It was explained that the initial discomfort was comparable to a deep tissue massage, but would subside with repeated application, resulting in a beneficial sensation [60,61]. The Numeric Rating Scale (NRS) (where 0 represents ‘no pain’ and 10 represents ‘worst possible pain’) was used for the subjective assessment of pain intensity to ensure that participants performed the rolling treatment at the desired controlled intensity. The participants were instructed to achieve a moderate intensity during the rolling treatment, corresponding to a value of 5 (first sequence) to 7 (second sequence) on the NRS. This value is within the range of therapeutic ‘acceptable discomfort’, which is considered necessary to release restrictions in the fascial tissue without inducing a reflexive muscular bracing [62].

The standardized routine for all twelve exercises resulted in a total duration of approximately 35 to 40 min per foam rolling session. Given the prescribed training frequency (immediately following the core cycling sessions), this led to a total weekly intervention volume of approximately 70 to 80 min of self-myofascial release. The applied foam rolling protocol corresponds in its total volume to the current scientific recommendations for optimizing flexibility and recovery [63]. Although studies less frequently report the number of repetitions than the total time, specifying 20 repetitions performed at a controlled speed results in an estimated rolling duration of 60 to 100 s per set. This timeframe falls ideally within the evidence-based range of 30 to 120 s per muscle group, which is described in systematic reviews as effective for the acute improvement of joint Range of Motion (ROM) and the reduction in muscle soreness (Delayed Onset Muscle Soreness, DOMS) [2]. Studies suggest that the positive effects on flexibility are significantly more pronounced with an application duration of over 60 s per muscle group [64]. Furthermore, the method follows clinical recommendations for treating Myofascial Trigger Points by including a targeted pause for several seconds and the execution of small, local rolling movements (3–5 cm) for specific reduction in muscle tone. Performing two sets per muscle group also falls within the common intervention range of one to three sets [21], which makes the protocol robust and scientifically sound in its structure and load.

**Figure 3:**
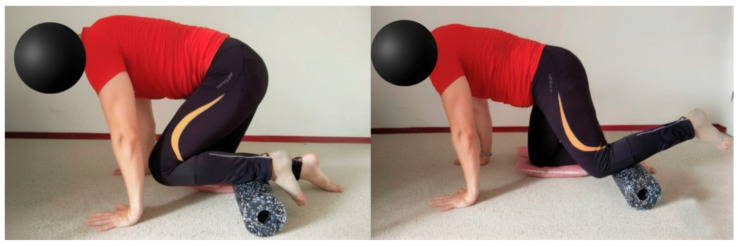
Initial and final phase of the exercise with the Blackroll^®^ for the tibialis anterior muscle (Source: own illustration).

In addition, participants were comprehensively informed about safety and hygiene measures. To prevent accidents and injuries, the training area had to be stable, non-slip, and free of sharp-edged objects, which is why the use of the gym mat was mandatory. The photo sequence in Figure 3 illustrates the initial and final phase of the exercise with the Blackroll^®^ for the calf muscles.

### 2.6. Operationalization

The main assumption of our study was that the use of the Blackroll^®^ foam roller increases mechanical performance and endurance in cycling. In the first hypothesis (H1), we tested the impact of foam rolling on maximal aerobic capacity in the form of maximal oxygen intake [65,66]. This pillar reflects the systemic “input” and the upper limit of aerobic metabolism, testing if SMR influences oxygen delivery or extraction. Because body mass influences both oxygen uptake and power output, VO_2_max is expressed relative to body weight (mL/min/kg), which allows for comparisons between different athletes.

The second hypothesis (H2) examined the impact of foam rolling on cardiovascular response measured as heart rate (heart beats per minute, bpm) at the aerobic and anaerobic lactate thresholds (2 mmol/L and 4 mmol/L). In our case, H2 assumes that foam rolling may positively impact the cardiovascular response, resulting in a lower heart rate at a constant lactate threshold [67]. A similar hypothesis with regard to myofascial release was already tested in [68], in which deep tissue massage therapy showed some impact on blood pressure and heart rate.

The third hypothesis (H3): Measuring lactate levels, therefore, indicates when an athlete transits from aerobic to anaerobic work [69]. In our case, we tested the impact of self-myofascial release on lactate levels between 100 and 250 watts. In H3 we took a closer look at the progression of the lactate curve between 100 and 250 watts and compared the mean slope of the lactate curve before and after the intervention. To analyze the lactate curve, we calculated the slope of the curve for the baseline test and post-test using the following formula:

(1) $$\mathrm{slope}\;=\;\frac{y_{2}-y_{1}}{x_{2}-x_{1}}$$

where *y*_1_ and *y*_2_ represent the lactate concentrations at the two workload levels and *x*_1_ and *x*_2_ correspond to 100 and 250 watts.

The final hypothesis (H4) analyzed the relationship between the application of the foam roller and mechanical performance and endurance. This included the assumption that the Blackroll^®^ has a positive impact on mechanical endurance, which we measured as power output relative to body weight (watt/kg) at the 2 and 4 mmol/L lactate thresholds. Here, we assumed that foam rolling increases the target variable and, therefore, improves performance and endurance. This represents the ultimate integration of cardiorespiratory capacity, metabolic efficiency, and mechanical economy.

As part of the descriptive analysis, we also examined anthropometric variables to describe the sample and how they evolved over the course of the six-month trial. This included the circumference of the left and right upper and lower legs (cm), body weight (kg) as well as the body mass index (BMI), expressed as weight in kilograms divided by height in meters squared.

### 2.7. Statistical Analysis

For statistical analysis we used the statistical software Python (version 3.1) and the statsmodel library. The statistical analysis for this study consists of two main steps. First, descriptive statistics were used to give an overview of the sample and the basic characteristics of the study participants. The primary objective of this analysis was to summarize the central tendencies and the variability of the data set. The demographic and anthropometric profile included the assessment of age, BMI and the circumferences of the upper and lower legs. Furthermore, the analysis encompassed a broad range of performance-related indicators. This included means and standard deviations (=*SD*) frequencies, central tendencies and variability for demographic and anthropometric indicators and variables related to performance and endurance such as physiological, metabolic and performance-specific variables. This approach ensured a systematic representation of all primary indicators relevant to the study’s scope.

Second, linear mixed-effects regression models (LMM) were developed and implemented to analyze the outcome variables. This modeling framework allows us to assess the within-subject changes over time (baseline vs. post-test) and between-group differences (intervention vs. control group). Moreover, the LMM includes a *group × time* interaction term, which captures the differential changes from baseline to post-test between the intervention and control groups and thus quantifies the effect of the intervention. While the intercept, group, time and the interaction term were specified as fixed effects, the individual IDs were modeled as random effects to control for inter-individual variability and the correlation of repeated measurements within individuals. Mathematically, the LLMs can be expressed in matrix notation as:

$$y_{i}=X_{i}\beta +Z_{i}b_{i}+\varepsilon_{i}$$

where *y_i_* denotes a vector containing the observations of the dependent variable for each subject, *X_i_* is an *n_i_* × *p* design matrix that includes the fixed effects for the independent variables, *β* represents a vector containing the regression coefficients for the fixed effects, *Z_i_* is an *n_i_* × *q* design matrix for modeling the random effects, *b_i_* is a vector of length *q* containing the coefficients for the random effects and *ε* represents the residual error term [70]. To assess the effect sizes for the mixed-effect models we calculated the marginal and conditional R-squared for each model.

To classify the magnitude of explained variance, we relied on the benchmarks established by Cohen where *R*^2^ ≈ 0.02 is considered small, *R*^2^ ≈ 0.13 medium, and *R*^2^ ≥ 0.26 large. In the context of mixed-effects modeling, these criteria are primarily applied to the marginal R^2^ to assess the practical significance of the fixed effects [71].

To ensure the validity of the analysis, model robustness was evaluated by testing the normality of the conditional residuals using a Shapiro–Wilk test and by visually inspecting Q–Q plots of the conditional residuals [72].

To ensure a rigorous evaluation of the intervention, statistical analyses were adjusted for age to account for its established influence on cardiovascular response and metabolic efficiency. Specifically, to address the observed baseline imbalance in age between the intervention and control groups, age was included as a fixed-effect covariate in all Linear Mixed Models (LMMs). This sensitivity analysis ensures that the observed changes are directly attributable to the foam rolling intervention rather than age-related physiological differences or declines in aerobic capacity.

This adjustment was particularly critical for isolating the intervention effect across our primary pillars: the systemic oxygen intake (VO_2_max relative to body mass), heart rate response at fixed lactate thresholds (2 and 4 mmol/L), and the progression of the lactate curve. Specifically, the slope analysis between 100 and 250 watts and the assessment of relative power output (W/kg) were adjusted to ensure that the observed improvements in mechanical endurance and metabolic economy were independent of the participants’ age, thereby increasing the precision of the estimated treatment effects.

## 3. Results

### 3.1. Descriptive Analysis

**Figure 4:**
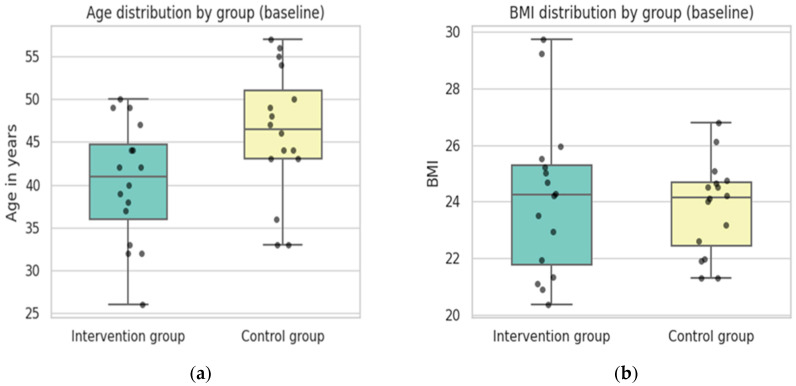
General description and overview of basic characteristics of the sample and anthropometric variables: (**a**) boxplots by group showing the age distribution; (**b**) boxplots showing the distribution of the body mass index by group. Colors indicate the respective groups, and dots represent outliers (Source: own illustration/Python).

At the beginning of the trial the sample had an average age of 43.19 years. As shown in Figure 4a, the intervention group and control group slightly differed in terms of age. Whereas the intervention group had a mean of 40.3 years with ages ranging from 26 to 50 years, the control group was older on average with a mean of 46.1 years ranging from 33 to 57 years. In summary, the control group was approximately six years older on average than the intervention group, which may be relevant if age influences study outcomes.

A lower degree of variability could be observed for BMI. At baseline, the overall BMI across both groups had a mean of 24 (*SD* = 2.2), ranging from 20.38 to 29.72. As can be seen in Figure 4b, most of the sample fell within the normal weight category. When split by group, the intervention group had a mean BMI of 24.12 (*SD* = 2.74) while the control group showed a mean of 23.82 (*SD* = 1.63). Both groups had nearly identical medians, although the intervention group had a wider spread (20.38–29.72) compared to the control group (21.32–26.79).

Post-test values indicated that BMI remained relatively constant in both groups. The calf and thigh circumferences were comparable between both groups at baseline. Post-test results showed only minor changes, which indicates that neither the exercises nor the intervention had a substantial effect on lower leg circumference. Similar patterns were observed for the thighs. Whereas the circumference of the left thighs averaged 52.76 cm in the intervention group at the baseline, the circumference in the control group was at 51.99 cm at the baseline—almost identical measurements for the right thighs. Over the course of the trial the average circumferences of the upper left leg in the intervention group slightly decreased, whereas the average circumference in the control group slightly increased, which could indicate some minor effects of the intervention on the upper legs, such as muscle tone changes, reduced fluid retention or minor adaptations in muscle morphology. However, the reverse pattern was observed for the right thigh, which contradicts the hypothesis that foam rolling had an impact on muscle tone or morphology.

Weight distribution also revealed no clear pattern. For the left side, both groups showed a minor decrease from baseline to post-test, while the right-side distribution followed different trends between groups. Overall, the descriptive statistics did not reveal major changes in leg circumferences or weight distribution from baseline to post-test, nor did they show consistent differences between the intervention and control groups over the course of the trial. (Table 3).

### 3.2. Analysis of the Hypotheses

#### 3.2.1. Maximal Oxygen Uptake (H1)

As shown in Table 4, the LMM sensitivity analysis revealed no significant difference between groups at baseline (*p* = 0.855), indicating successful randomization. Within the control group, there was a non-significant trend toward improvement over time (*p* = 0.085). Regarding the primary hypothesis, the *group × time* interaction, representing the additional gain in the intervention group, remained positive (*β* = 1.404) but did not reach statistical significance (*p* = 0.234) after adjusting for age.

The model’s variance components provided critical insights into the data structure. A key finding of the sensitivity analysis was the significant impact of *age* on VO_2_max levels (*β* = −0.342, *p* = 0.006), confirming it as a relevant covariate. The marginal *R*^2^ increased to 0.124, suggesting that the inclusion of age, alongside the experimental factors (*group* and *time*), explained 12.4% of the total variance. In contrast, the conditional *R*^2^ was 0.885, demonstrating that 88.5% of the variance was accounted for when considering individual differences and age.

The results suggest that while the intervention group showed a nominally higher increase in VO_2_max compared to the control group, the effect was not statistically significant, even when accounting for the age-related decline in aerobic capacity. The substantial disparity between the marginal and conditional *R*^2^ values highlights that the physiological “baseline” and individual characteristics, particularly age, exerted a far greater influence on the outcomes than the intervention itself. Specifically, the high stability of VO_2_max levels within subjects suggests that the individual physiological profile remained dominant despite the treatment.

**Figure 5:**
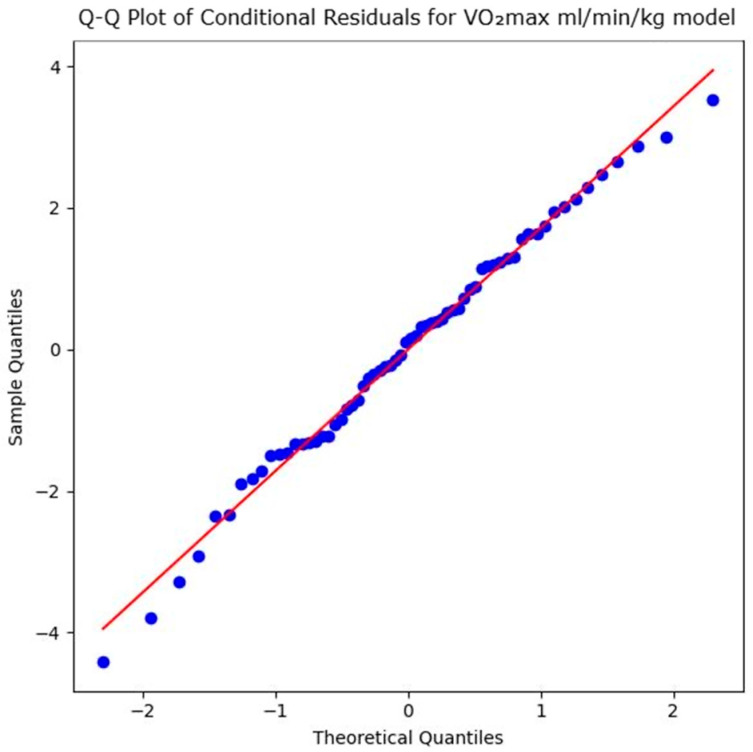
Q–Q plot of conditional residuals for maximal oxygen uptake in mL/min/kg model. Blue dots represent the observed residuals, and the red line indicates the theoretical normal distribution (Source: own illustration/Python).

The model assumptions of the LLM to evaluate the impact of the intervention on the VO_2_max in mL/min/kg were verified via a Shapiro–Wilk test on the conditional residuals, which indicated no significant deviation from normality (*W* = 0.9905, *p* = 0.906). As the Q–Q plot of the conditional residuals in Figure 5 shows, the observed data points closely follow the diagonal reference line, indicating that the residuals are approximately normally distributed. This confirms the appropriateness of the LMM and the reliability of the standard error estimates.

**Figure 6:**
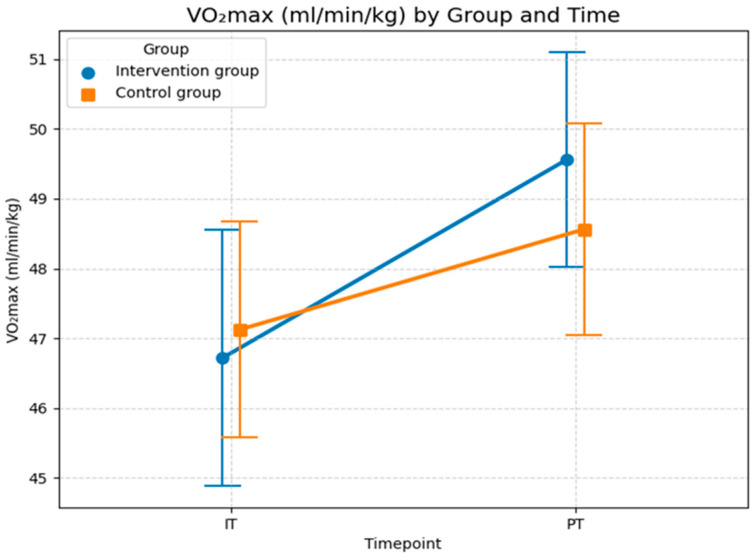
Maximal oxygen uptake for both groups and both time points (IT = initial test at the beginning; PT = post-test after 6 months). Values are means ± SD. (Source: own illustration/Python).

Although the LMM did not show a statistically significant effect of foam rolling on oxygen uptake, the graph in Figure 6 illustrates changes in the means and standard deviations for both groups across both time points. In the initial test, the control group started with a slightly higher mean (47.1) than the intervention group (46.7). In the post-test, both groups showed improvement, but the intervention group achieved descriptively a larger increase, reaching a mean of 49.6 (an increase of 2.8), thereby surpassing the control group (mean = 48.6, an increase of 1.4). The standard deviation (*SD*) for the intervention group decreased from 7.3 to 6.2, indicating more homogeneous performance. This suggests that maximal oxygen uptake improved in relative terms in the intervention group. This resulted in a steeper upward trajectory in the intervention group compared to the control group. While the descriptive statistics indicate an effect of foam rolling on maximal oxygen uptake, the non-significant interaction (*p* = 0.234) in the sensitivity-adjusted model confirms that this difference cannot be reliably attributed to the intervention. This is likely due to the limited sample size and the high inter-individual variability and demographic factors (age), as reflected in the high conditional *R*^2^ value (0.885).

#### 3.2.2. Heart Rate at the Aerobic and Anaerobic Threshold (H2)

The second hypothesis (H2) assumes that foam rolling has a positive effect on heart rate measured in heart beats per minute (bpm) at the aerobic and anaerobic lactate thresholds (2 mmol/L and 4 mmol/L). The statistical analysis in Table 5 revealed no significant interaction effect between group and time (*β* = −0.375; *SE* = 2.436; *z* = −0.154; *p* = 0.878) after adjusting for age. This indicates that the development of heart rate at the 2 mmol/L threshold in the intervention group did not differ significantly from that of the control group over the study period and indicates that the intervention did not produce a measurable shift in this physiological parameter compared to the control group. Furthermore, the main effects for group (*β* = 1.063; *p* = 0.774) and time (*β* = 0.875; *p* = 0.611), and age (*β* = −0.125; *p* = 0.452) were non-significant. Consequently, there were no meaningful baseline differences between groups, nor was there a general change in heart rate over time across the entire sample which suggests that neither group experienced a general improvement in cardiovascular economy.

A central finding of the model is the variance distribution. The marginal *R^2^* was only 0.007, illustrating that the experimental factors (group and time) and the covariate age possess almost no explanatory power for the total variance in heart rate. In contrast, the conditional *R*^2^ was 0.789. This high value is almost entirely attributable to the inclusion of random effects (between-group variance: 86.12; residual variance: 23.73). This demonstrates strong inter-individual constancy: heart rate values are highly person-specific, with approximately 78.9% of the observed variance explained by individual differences between subjects. The stark contrast between the marginal *R*^2^ and the conditional *R*^2^ reveals that the vast majority of the variance is explained by the participants’ unique baseline characteristics rather than the experimental treatment or demographic factors like age.

**Figure 7:**
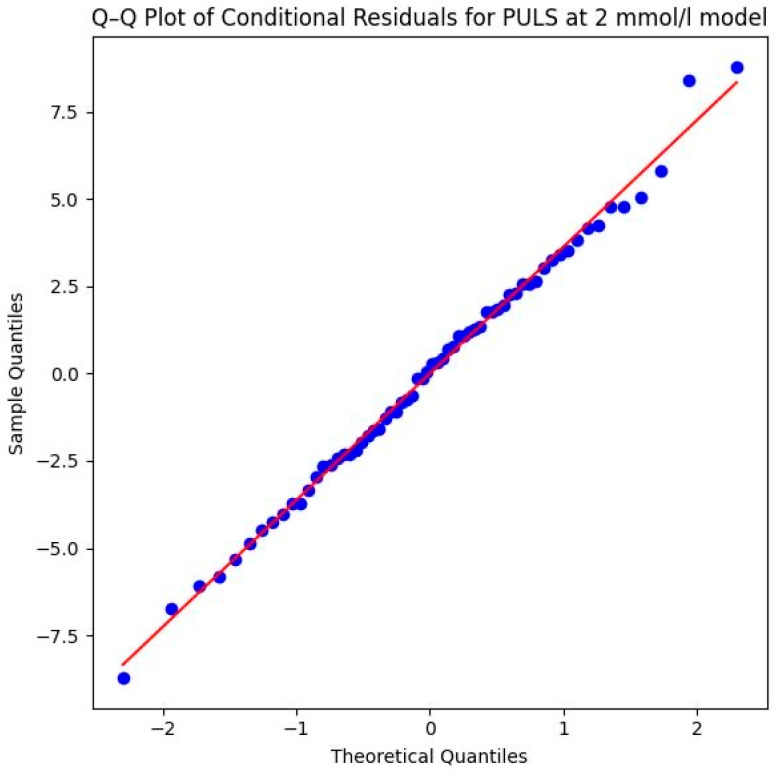
Q–Q plot of conditional residuals for PULS model at 2 mmol/L lactate level. Blue dots represent the observed residuals, and the red line indicates the theoretical normal distribution (Source: own illustration/Python).

Model assumptions were verified through both numerical and visual assessments. The Shapiro–Wilk test of the conditional residuals yielded *W* = 0.995 (*p* = 0.997), indicating a normal distribution. This is visually confirmed by the Q–Q plot shown in Figure 7, where the sample quantiles align almost perfectly with the theoretical normal distribution line, showing no significant skewness or outliers. This confirms the statistical validity of the calculated *p*-values.

**Figure 8:**
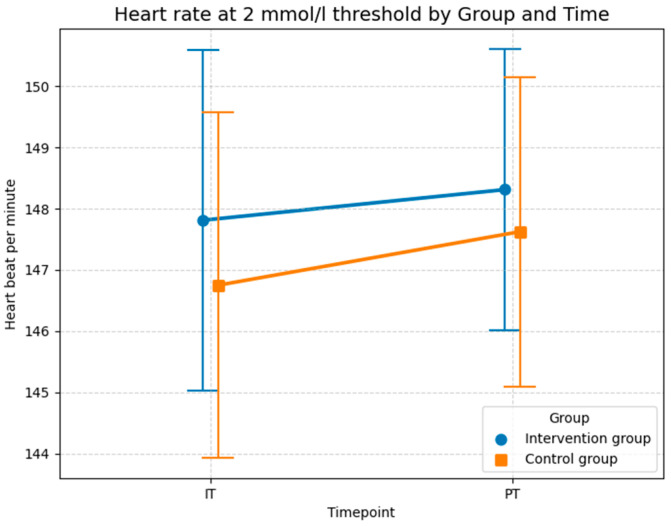
Descriptive statistics (means and SD) of the heart rate measured in beats per minute for both groups and both time points at the 2 mmol/L lactate level (Source: own illustration/Python).

The descriptive statistics presented in Figure 8 are consistent with the results of the LMM analysis, showing no substantial differences between the groups. The average heart rate increased in both groups from baseline test to post-test. In the control group, the mean heart rate increased slightly from 146.8 bpm (*SD* = 11.3) in the initial test to 147.6 bpm (*SD* = 10.1) at post-test. A similar minor increase was observed in the intervention group, where the heart rate rose from 147.8 bpm (*SD* = 11.1) in the initial test to 148.3 bpm (*SD* = 9.2) at the post-test. As illustrated in Figure 8, the increase in heart rate was slightly greater in the control group (+0.8 bpm) than in the intervention group (+0.5 bpm), which could indicate a minor effect of foam rolling on improving the respiratory response at the 2 mmol/L threshold. Like the analysis of maximal oxygen uptake, the LMM results in Table 5 suggest that this effect is not statistically significant, likely due to the limited sample size and the high inter-individual variability, as evidenced by the high conditional *R*^2^ value (0.789).

In addition to the heart rate at the aerobic level, Table 6 displays the results of the LMM analyzing heart rate at the anaerobic level measured at the 4 mmol/L lactate threshold. The LMM started with an intercept of 170.438 beats per minute for the control group. A comparison between the groups at the start of the study showed no significant differences (*p* = 0.791), indicating successful randomization of the 32 subjects.

When examining the development over time, the control group showed only a minimal, non-significant increase in heart rate of 0.750 beats per minute (*p* = 0.232). The decisive measure for the success of the study, the interaction effect between group and time, was also non-significant with a coefficient of −0.750 (*p* = 0.398) after adjusting for age. The main effect for age itself was non-significant (*β* = −0.118, *p* = 0.485), suggesting that age did not exert a dominant influence on the heart rate at this specific threshold in this sample. Consequently, the intervention had no statistically detectable influence on the heart rate at the anaerobic threshold; the changes in the intervention group did not differ significantly from those in the control group.

The examination of the variance components clarifies the structure of the data: the model has a marginal *R*^2^ of only 0.004, meaning that the fixed effects (intervention and time) explain only about 0.4% of the total variance. In stark contrast, the conditional *R*^2^ is 0.969. This extremely high value shows that nearly 97% of the variance is explained by individual differences between the subjects (Residual Variance = 97.148). The heart rate at this level is thus a highly individual physiological characteristic that remains stable over time but was not significantly shifted by the investigated intervention.

Despite the methodological stability, the results must be viewed critically. Dominance of Individual Variance: The extremely high proportion of subject variance (approx. 97%) suggests that the heart rate at the 4 mmol/L threshold is a physiologically deeply anchored value in this sample. This makes it significantly harder to prove training effects, as individual profiles effectively mask any intervention effects. The high value of the Group Variance compared to the low error term (Scale: 3.150) confirms the measurement precision within individuals but simultaneously underlines that variability between subjects is the main obstacle to making generalizable statements about the effectiveness of the intervention for this specific heart rate parameter.

**Figure 9:**
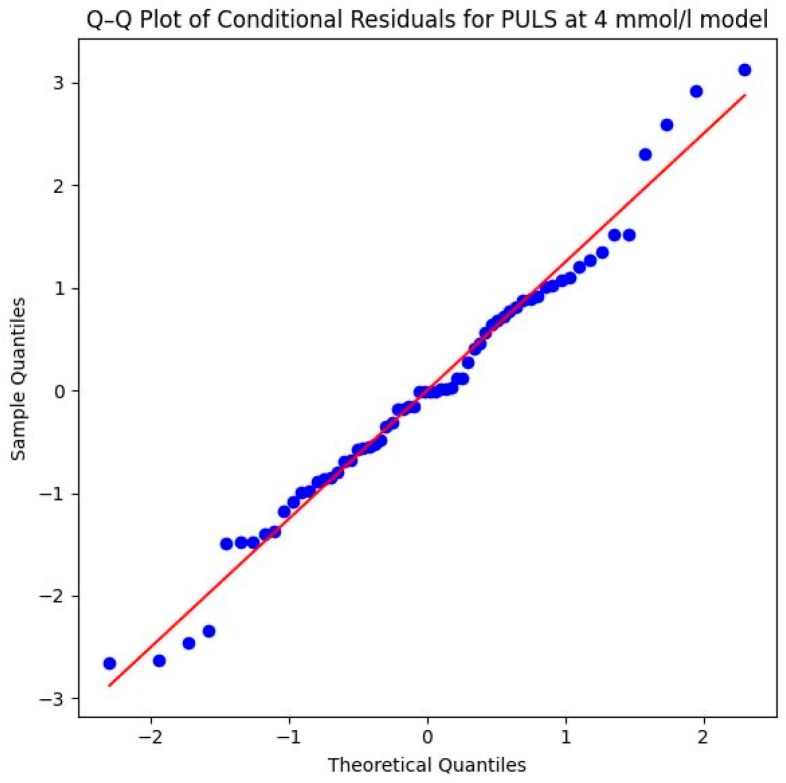
Q–Q plot of conditional residuals for PULS model at 4 mmol/L lactate level. Blue dots represent the observed residuals, and the red line indicates the theoretical normal distribution (Source: own illustration/Python).

Methodologically, the model is very robust. The successful convergence and the Shapiro–Wilk test (*W* = 0.9821, *p* = 0.4784) confirm that the residuals are normally distributed. This assumption is visually supported by the Q–Q plot in Figure 9 of the conditional residuals, the data points follow the diagonal reference line very closely, which indicates an excellent fit to the theoretical normal distribution and ensures the statistical validity of the calculated *p*-values.

**Figure 10:**
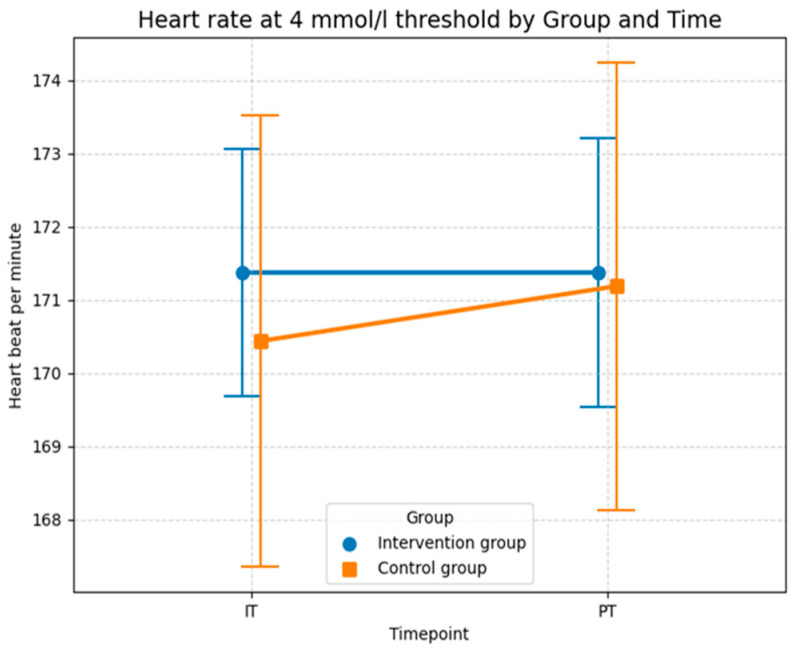
Descriptive statistics (means and SD) of the heart rate measured in beats per minute for both groups and both time points at the 4 mmol/L lactate level (Source: own illustration/Python).

Consistent with the descriptive statistics for heart rate at the aerobic level, the curves for heart rate at the anaerobic level in Figure 10 show that the control group experienced a greater increase compared to the intervention group. In the control group, the mean heart rate was 170.4 bpm (*SD* = 12.33) in the initial test and increased slightly to 171.2 bpm (*SD* = 12.2) at the post-test. The intervention group recorded a mean heart rate of 171.4 bpm (*SD* = 6.8) at the initial test. Notably, this value remained almost constant at 171.4 bpm (*SD* = 7.3) at the post-test. Overall, the mean values of all measurements were very similar, although the standard deviation in the intervention group was notably lower than in the control group. While the lower standard deviation and constant HR in the intervention group could descriptively suggest improved cardiovascular efficiency, the LMM results in Table 6 confirm that this effect is not statistically reliable, probably due to the small sample size and the overwhelming influence of individual variability, as reflected by the conditional *R*^2^ of 0.969.

#### 3.2.3. Lactate Curves (H3)

Hypothesis H3 investigates the impact of foam rolling on the slope of the lactate curve from 100 to 250 watts with workload increasing by 50 watts every three minutes. The comparison focuses on how the lactate curve develops in both groups before and after the intervention. As shown in Table 7, the statistical evaluation of the lactate curve slope using an LMM provides clear evidence of the effectiveness of the intervention, even after adjusting for age. At the start of the study (Baseline), the control group had an average slope of 0.024 (represented by the Intercept). This initial difference between the groups was not statistically significant (*p* = 0.403), confirming a comparable starting point for the 32 subjects.

Over the course of the study, the control group showed almost no change, with a non-significant time effect (*p* = 0.644), ending at a value of 0.023. In contrast, the intervention group recorded a significant improvement, due to the highly significant interaction effect of −0.007 (*p* = 0.004), the slope of the lactate curve decreased substantially. This flattening of the curve is a clear indicator of increased metabolic efficiency, as the lactate concentration per additional watt of power increased significantly less after the training period. Notably, age did not have a significant influence on the slope (*β* = 0.0001, *p* = 0.712), further isolating the intervention as the primary driver of change.

The quality of the model underscores the relevance of these results. The marginal *R^2^* of 0.145 indicates that the experimental factors (group and time) and age explain approximately 14.5% of the total variance, a substantial effect size for physiological adaptations. The conditional *R*^2^ of 0.812 further confirms that the overall model accounts for over 81% of the data variability.

The results demonstrate that the lactate slope is a significantly more sensitive marker for physiological adaptation to this specific intervention compared to heart rate at a fixed lactate level. While individual differences continue to play a role, the intervention was able to systematically improve the metabolic response to increasing workloads. The stability of the control group, combined with the robustness of the effect in the sensitivity analysis, allows for the conclusion that the observed increase in performance is directly attributable to the training protocol with the Blackroll^®^ rather than random fluctuations or age-related differences.

**Figure 11:**
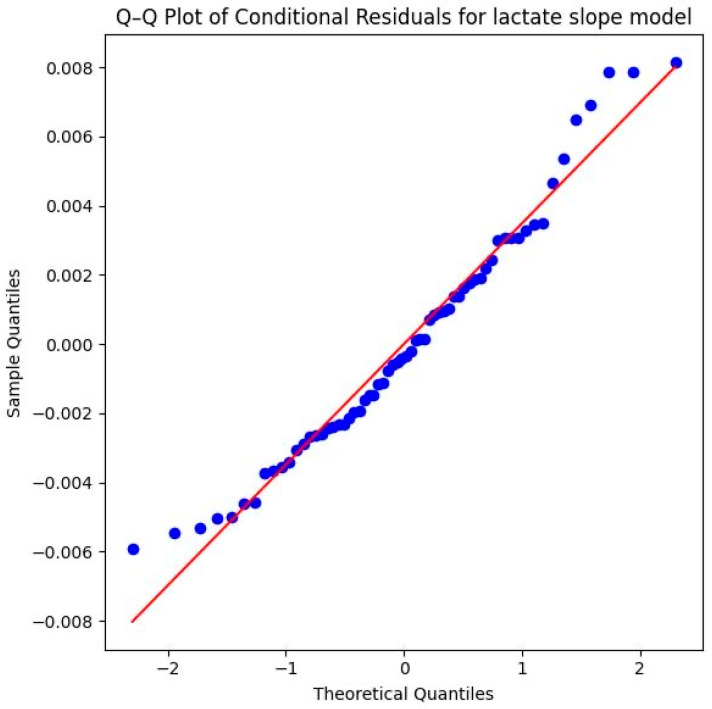
Q–Q plot of conditional residuals for the lactate slope model. Blue dots represent the observed residuals, and the red line indicates the theoretical normal distribution (Source: own illustration/Python).

Methodologically, the model is very well-supported. The Shapiro–Wilk test (*W* = 0.9661, *p* = 0.0756) as well as the provided Q–Q plot of the conditional residuals demonstrate that the residuals are normally distributed. The data points follow the diagonal reference line closely in the plot, which underscores the statistical reliability of the significant interaction effect (Figure 11).

**Figure 12:**
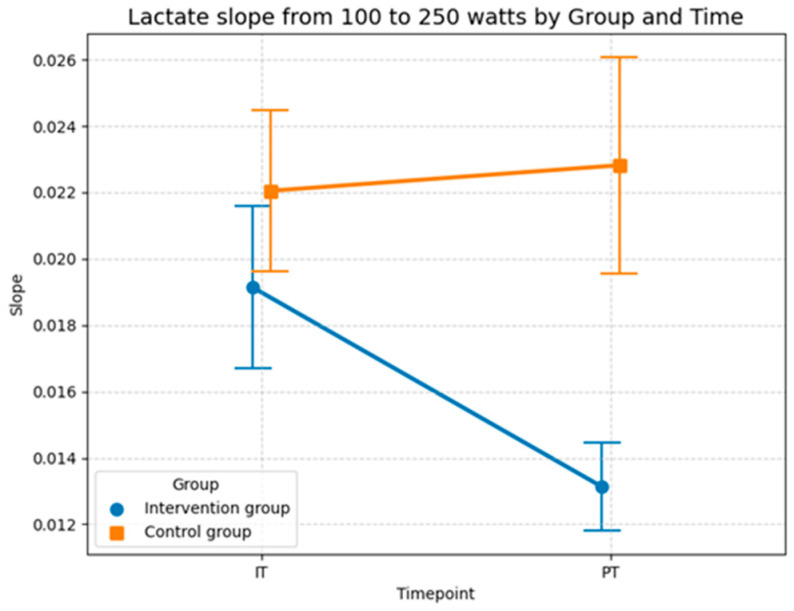
Descriptive statistics (means and SD) of the slopes of the lactate curves from 100 to 250 watts performance output for both groups and both time points (Source: own illustration/Python).

The descriptive statistics of the lactate slopes between 100 and 250 watts provide further evidence for the intervention effect observed in the LLM model. In the control group, the mean slope remained nearly unchanged from baseline to post-test (0.0220 (*SD* = 0.0097) to 0.0228 (*SD* = 0.013)), suggesting no meaningful adaptation in lactate accumulation with increasing workload. In contrast to the control group, the intervention group showed a clear reduction in slope values across time, with the mean decreasing from 0.0191 (*SD* = 0.0098) at the baseline to 0.0131 (*SD* = 0.0053) at the post-test. This indicates a flatter lactate curve progression, reflecting a slower rise in lactate levels at higher workloads, which indicates an improved endurance performance. In sum, these descriptive findings align with the LMM results (Table 7) and highlight that the intervention group benefited from a more favorable metabolic adaptation, systematically improving the response to increasing workloads compared to the control group (Figure 12).

#### 3.2.4. Power Output at the Aerobic and Anaerobic Threshold (H4)

Hypothesis H4 tests the relationship between foam rolling and performance measured in watts per kg at different lactate thresholds. The statistical investigation of relative power output at the 2 mmol/L lactate threshold in Table 8 demonstrates a significant improvement in aerobic performance resulting from the intervention, even after adjusting for age. The model established a baseline intercept of 2.666 W/kg, representing the starting value for the control group. At the start of the study, the comparison between the control group and the intervention group showed no significant difference (*p* = 0.849). This confirms a homogeneous baseline across the 32 subjects, ensuring that any subsequent changes can be attributed to the experimental conditions rather than initial performance gaps.

Throughout the study, both groups showed development, though the increase in the intervention group was markedly more pronounced. While the control group showed a modest increase over time (*p* = 0.032), the intervention group achieved a significantly higher improvement driven by a robust interaction effect of 0.145 (*p* = 0.031). Interestingly, age showed a significant negative correlation with relative power (*β* = −0.012, *p* = 0.024), indicating that younger participants generally started at a higher performance level. However, the significance of the interaction effect (*Group × Time*) remains stable, proving that the intervention effect is independent of the participants’ age. Further this finding illustrates that the specific intervention program improved performance at the aerobic threshold beyond the extent of natural temporal fluctuations or mere habituation effects.

The statistical quality of the model is supported by a conditional *R*^2^ of 0.954, which indicates that over 95% of the total variance is explained by the model. Although the marginal *R*^2^ increased to 0.065 (explaining 6.5% of the variance through group, time, and age), the effect remains statistically robust due to the high precision of the measurements.

The results show that relative power (watt/kg) at 2 mmol/L is a highly reliable and sensitive marker for capturing training effects. In contrast to pure heart rate parameters, this value reflects actual mechanical performance at the metabolic threshold. While the high person-specific stability (high random intercept variance of 0.325 compared to a low residual variance of 0.018) indicates that the individual fitness level remains the largest source of variance, the intervention was nevertheless able to raise this level systematically and demonstrably across the age spectrum of the sample.

**Figure 13:**
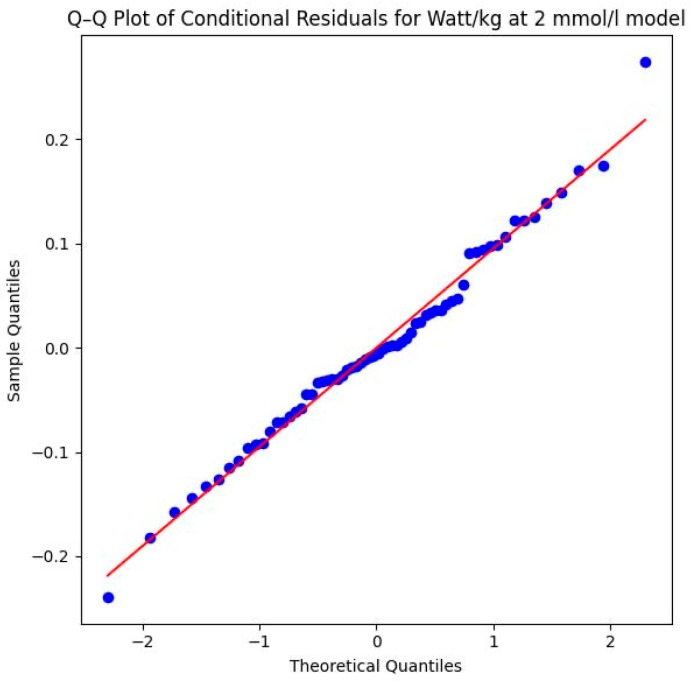
Q–Q plot of conditional residuals for the watts per kg model at the aerobic level. Blue dots represent the observed residuals, and the red line indicates the theoretical normal distribution (2 mmol/L threshold) (Source: own illustration/Python).

Methodological validity is further confirmed by the Shapiro–Wilk test (*W* = 0.9898, *p* = 0.8788) and the Q–Q plot of the conditional residuals. In the plot illustrated in Figure 13, the data points follow the diagonal reference line almost perfectly. This proves that the model assumptions are excellently met and the calculated *p*-values are highly reliable.

**Figure 14:**
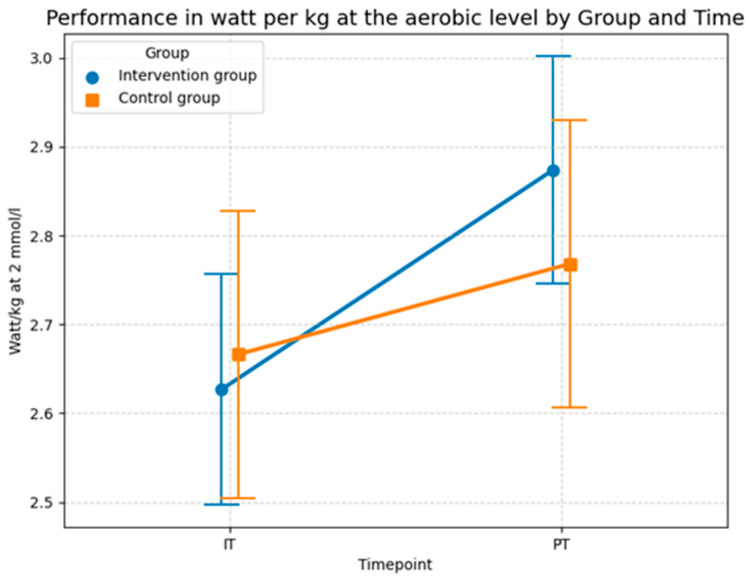
Descriptive statistics (means and SD) of the performance in watts per kg at the aerobic level for both groups and both time points (Source: own illustration/Python).

The descriptive statistics of the performance output at the aerobic level in Figure 14 aligns with the results of the LMM model and shows that performance increased in both groups, although the intervention group experienced a greater improvement compared to the control group. In the control group the mean power improved from 2.67 watts per kg (*SD* = 0.65) in the initial test to 2.77 watts per kg (*SD* = 0.65) at the post-test. The intervention group showed a more substantial increase in performance, where the mean value rose from 2.63 watts per kg (*SD* = 0.52) at the initial test to 2.87 watts per kg (*SD* = 0.51) at the post-test. Thus, both groups recorded an increase in aerobic power, with the absolute gain in the intervention group (0.24 watts/kg) being more than double the gain in the control group (0.10 watts/kg). This stronger improvement in the intervention group is consistent with the significant interaction effect observed in the sensitivity-adjusted LMM (Table 8) and is most likely attributable to the specific intervention program, as the model confirms that this progress remains robust even when accounting for individual differences and age (*p* = 0.031).

In addition to the performance at the aerobic level, the LMM model in Table 9 analyzes the impact of foam rolling on the performance in watt per kg at the anaerobic level at the 4 mmol/L lactate threshold. The statistical investigation demonstrates a highly significant improvement in aerobic performance resulting from the foam rolling intervention, even after adjusting for age. The model established a baseline intercept of 3.820 W/kg, representing the initial level for the control group. At the beginning of the study, the comparison between the control group and the intervention group showed no significant difference (*p* = 0.760). This confirms a homogeneous baseline across the 32 subjects, ensuring that subsequent changes were the result of the experimental conditions rather than initial performance gaps.

Throughout the study, both groups showed development, however, the improvement in the intervention group was considerably more pronounced. While the control group experienced a significant increase over time (*p* = 0.022), the intervention group achieved a much stronger gain, evidenced by a highly significant interaction effect of 0.256 (*p* = 0.007). The covariate age was identified as a significant negative predictor of performance (*β* = −0.015, *p* = 0.012), yet its inclusion did not diminish the intervention effect. This finding indicates that foam rolling contributed to enhanced performance at the anaerobic threshold, allowing participants to sustain a higher watt output per kg at 4 mmol/L lactate regardless of their age.

The model’s explanatory power is substantial, with a conditional *R*^2^ of 0.902, indicating that over 90% of the variance is accounted for by the model. The marginal *R*^2^ increased to 0.115, showing that approximately 11.5% of the total variance is directly attributable to the fixed factors of group, time, and age. The results suggest that watt/kg at 4 mmol/L is an exceptionally sensitive marker for the physiological adaptations induced by foam rolling, with the high significance level of the interaction effect (*p* = 0.007) suggesting a strong impact on high-intensity aerobic capacity.

**Figure 15:**
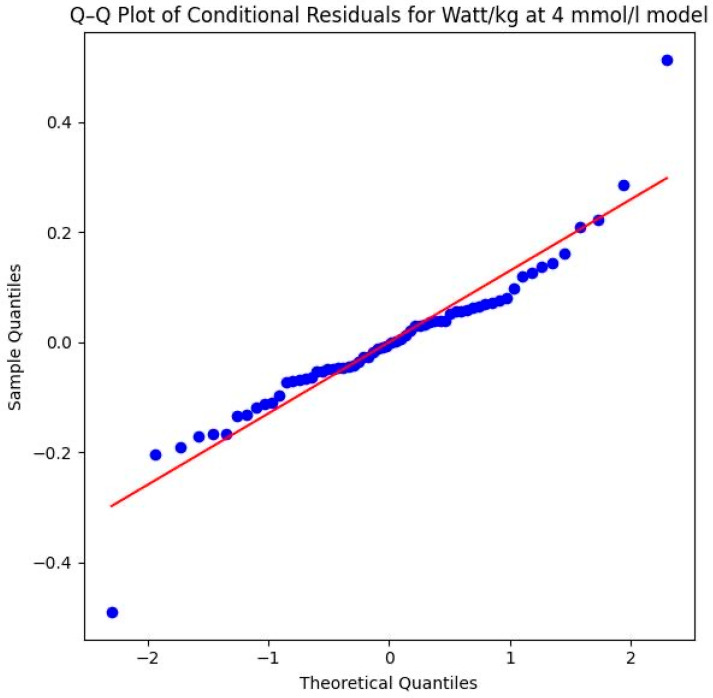
Q–Q plot of conditional residuals for the watts per kg model at the anaerobic level. Blue dots represent the observed residuals, and the red line indicates the theoretical normal distribution (4 mmol/L threshold) (Source: own illustration/Python).

The Shapiro–Wilk test yielded a *p*-value of 0.0005 (*W* = 0.9209), which indicates that the residuals deviate from a perfect normal distribution. This deviation is further confirmed by visual evidence in the Q–Q plot in Figure 15. The plot reveals outliers at both the lower and upper theoretical quantiles that pull away from the diagonal reference line. While the majority of the data points follow the trend, these deviations at the extremes suggest that the assumption of normality is not fully met, which should be considered when interpreting the precision of the significance levels for this specific parameter. However, the violation of the normality assumption (Shapiro–Wilk *p* < 0.05) and the observed outliers in the Q–Q plot suggest that some individuals may have responded differently to the intervention, or that the high-intensity nature of the 4 mmol/L threshold introduces more physiological “noise” compared to the more stable 2 mmol/L threshold. Despite these violations, LMMs generally remain robust. The highly significant *p*-value (*p* = 0.007) therefore lends further support to the credibility of the systematic performance increases.

**Figure 16:**
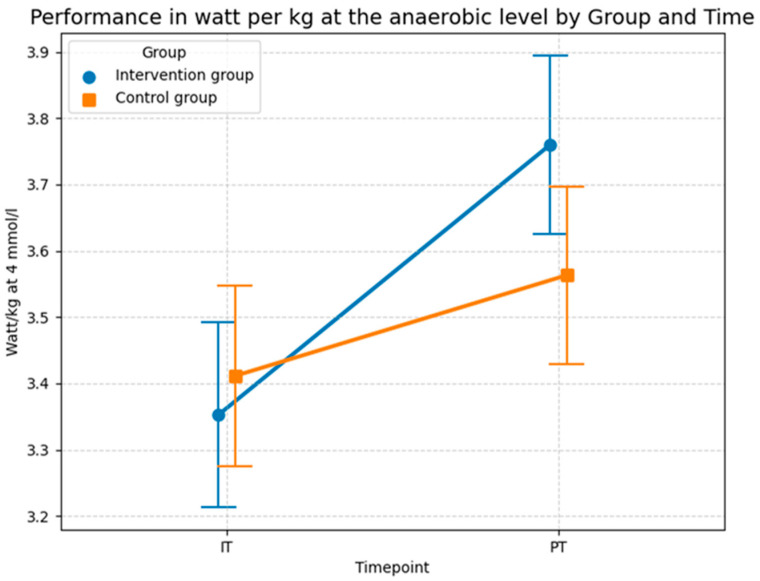
Descriptive statistics (means and SD) of the performance in watts per kg at the anaerobic level for both groups and both time points (Source: own illustration/Python).

The descriptive statistics in Figure 16 generally confirm the results of the LMM in Table 9. The intervention group showed a stronger improvement in terms of performance than the control group at the anaerobic level. While the performance of the intervention group increased from 3.353 watts per kg (*SD* = 0.56) during the baseline test to 3.76 watts per kg (*SD* = 0.54) at the 4 mmol/L lactate threshold during the post test, average performance of the control group only increased from 3.411 watts per kg (*SD* = 0.54) to 3.563 watts per kg (*SD* = 0.54). In conclusion, both groups increased their anaerobic power. However, the absolute gain in the intervention group (0.41 watts/kg) was substantially higher than in the control group (0.15 watts/kg). Evidently, this program was more effective than the standard program of the control group at increasing athletic performance, particularly anaerobic capacity, as confirmed by the highly significant interaction effect (*p* = 0.007) in the sensitivity-adjusted model. 

## 4. Discussion

The goal of this RCT was to evaluate the long-term effects of foam rolling as an SMR technique on performance- and endurance-related outcomes among road race cyclists. Based on theoretical frameworks from mechanical, neurological, physiological or psychophysiological perspectives, we hypothesized that post-exercise foam rolling would positively influence maximum aerobic performance, lactate thresholds as an indicator of submaximal training performance, and mechanical performance. As our analysis showed, however, the conclusions that can be drawn from our empirical results are to some degree inconclusive, which reflects the broader inconsistencies in scholarly literature. While some outcomes indicate potential benefits of foam rolling, other results suggest limited or no effects—a problem that highlights the complexity of linking SMR, performance and endurance.

Regarding cardiorespiratory capacity, our analysis could not confirm our first hypothesis, in which we assumed that foam rolling may have a positive effect on oxygen uptake. Although descriptive statistics showed that on average oxygen uptake improved more strongly in the intervention group than in the control group, the LMM model did not find statistically significant differences between the intervention and control group. Although the intervention group showed a descriptively stronger increase in VO_2_max (+2.9 mL/min/kg vs. +2.5 mL/min/kg in the control group), this result must be regarded as a mere trend without statistical confirmation due to the exploratory nature of the study and the limited power (72.4%). The risk of a Type II error implies that moderate effects may exist but could not be unequivocally detected in this setting.

Our sensitivity analysis confirmed that even when controlling for the significant influence of age on maximal oxygen uptake (*p* = 0.006), no treatment effect emerged. This suggests that the age disparity, while physiologically relevant for absolute baseline values, did not mask a potential intervention effect, as both groups remained stable in their systemic markers. This reinforces the view that localized SMR does not reach the threshold of central cardiovascular stimulation required to alter VO_2_max or submaximal heart rate, regardless of the athlete’s age-related baseline. Physiologically, the age-related decline in VO_2_max is primarily driven by a reduction in maximal cardiac output and an uneven distribution of blood flow, which limits oxygen delivery to the working muscles until late middle age. Furthermore, the oxidative capacity of skeletal muscle tends to decrease with age, partly due to mitochondrial dysfunction, which further sets a physiological ‘ceiling’ that localized myofascial interventions are unlikely to overcome [73].

From a theoretical point of view, this challenges mechanical and physiological explanations that argue that SMR positively influences oxygen delivery through tissue compliance or increased blood flow to the capillaries. The findings are consistent with potential improvements in metabolic and mechanical efficiency, although the specific physiological mechanisms remain speculative, as parameters such as microcirculation or muscle stiffness were not directly measured. It should be acknowledged that cardiovascular exercise remains the primary driver for robust vascular adaptation and systemic blood flow. Foam rolling should therefore be viewed as a supplemental tool rather than a replacement for aerobic activity, serving specifically to enhance localized perfusion and myofascial recovery. This result is consistent with a study by Stroiney et al., who found no effect of self-myofascial release on maximal oxygen consumption [74]. This aligns with the established physiological understanding that in highly trained individuals, VO_2_max is primarily limited by the central cardiovascular system’s capacity to deliver oxygenated blood to the periphery, rather than by localized muscular oxygen extraction or tissue compliance [2,75].

A similar conclusion can be drawn on cardiovascular efficiency, which we tested in H2, using submaximal heart rate at the aerobic (2 mmol/L) and anaerobic (4 mmol/L) thresholds. While the descriptive statistics show some minor improvements in terms of heart rate in the intervention group compared to the control group, especially at the anaerobic level, the LMMs failed to demonstrate a clear benefit of foam rolling for the cardiorespiratory efficiency and, therefore, could not confirm theories suggesting that myofascial release techniques would result in lower heart rates at constant workloads. The high inter-individual constancy observed in our data (Conditional *R*^2^ up to 0.969) suggests that heart rate at these intensities is a physiologically anchored value that remains remarkably resistant to localized tissue interventions [45]. Furthermore, the higher average age in the control group likely limited cardiovascular plasticity and adaptation. Longitudinal data from Fleg et al. [76] show that VO_2_peak decline is not constant but accelerates significantly after age 40, regardless of activity levels. This decline is primarily driven by a reduced oxygen pulse, while maximal heart rate remains more stable (4–6% decline per decade) [76]. These physiological trajectories suggest that the six-year age gap in our cohort is a relevant factor for baseline performance and adaptive capacity, potentially masking minor group differences despite statistical controls.

In contrast to these systemic cardiorespiratory markers, our analysis revealed more compelling evidence on metabolic kinetics (H3). Here, we found evidence that myofascial release might reduce the slope of the lactate curve. While the slope of the lactate curve in the control group essentially remained the same, the intervention group showed a statistically significant decrease, which indicates that foam rolling delayed the accumulation of lactate and, thus, increased endurance. Notably, the significance of the flattened lactate curve (*p* = 0.004) persisted after adjusting for age. This is a crucial finding, as older athletes typically exhibit slower lactate clearance and reduced buffering capacity [77]. By identifying the intervention as the primary driver of the improved slope, we can conclude that the metabolic benefits of SMR are robust enough to overcome the physiological disadvantages associated with the slightly higher age profile of the control group.

This improvement in metabolic kinetics is consistent with theoretical models of improved lactate clearance. Although not directly measured, research by Hotfiel et al. [14] and Alonso-Calvete et al. [15] demonstrated a significant increase in arterial tissue perfusion, vessel diameter, and blood flow velocity immediately following SMR interventions. Theoretically, this increased volume flow enhances the transport capacity for metabolic byproducts out of the working musculature, providing a mechanical basis for a steeper decline in the lactate curve (lactate slope). These mechanistic insights are reflected in metabolic outcomes. Laffaye et al. showed that an SMR treatment of at least 120 s led to significantly faster lactate clearance and lower perceived pain 30 min post-exercise compared to passive recovery [78]. Furthermore, Kasahara et al. confirmed that blood lactate concentrations were significantly reduced by SMR following high-intensity exercise, a process that also correlated with a faster recovery of executive functions [19,24]. Other recent evidence from 2025 supports these proposed pathways as Alansari et al. demonstrated via thermal imaging and biochemical markers that SMR significantly enhances lactate reduction and normalizes muscle temperature more effectively than passive recovery [16].

In sum, this aligns with mechanical and neuromuscular explanations of SMR stating that reduced fascial stiffness and a shift in the thixotropic state of the extracellular matrix may facilitate lactate metabolism and increase endurance and performance [10,11,12]. The optimization of lactate kinetics may be attributed to potential enhanced fluid dynamics within the tissue. Rather than a simple acceleration of clearance, the reduced fascial viscosity likely alleviates interstitial congestion, thereby streamlining the transport of metabolites into the vasculature [18,19]. Optimization of lactate kinetics, primarily managed by Type I muscle fibers, represents a crucial adaptation for submaximal endurance, allowing athletes to sustain higher power outputs before reaching critical fatigue [26,79].

Another hypothesis that could be confirmed was H4, in which analyzed the impact of self-myofascial release using a foam roller on the mechanical performance measured in watts per kg at the aerobic and anaerobic level. At both lactate thresholds (2 mmol/L and 4 mmol/L) our LMMs showed that the intervention group improved more substantially in watts per kg over time than the control group, indicating that foam rolling positively affects the mechanical performance, although the significance of the improvement was higher at the anaerobic level than at the aerobic level. The intervention had a 76% larger effect on performance at the anaerobic threshold than at the aerobic threshold. While our model identified age as a significant negative predictor for power output at both thresholds (*p* = 0.024 and *p* = 0.012), the intervention effect remained highly significant (*p* = 0.007 at 4 mmol/L). This suggests that while age sets the absolute performance ceiling, the relative gain in mechanical efficiency through SMR is achievable across different age brackets. Aging is inherently associated with increased fascial thickness and reduced tissue elasticity [5], which elevates thixotropic resistance, the internal energy required to move limbs against tissue friction. By inducing a potential thixotropic shift in the extracellular matrix, SMR might counteract age-dependent mechanical constraints [5,6], although this remains a theoretical framework in the context of the present results.

Regarding this hypothesis (H4), the improvements observed can be attributed to an increased electromyographic fatigue threshold [10]. By stimulating mechanoreceptors within the fascial network, specifically Ruffini and Pacini corpuscles, SMR is hypothesized to optimize muscle recruitment patterns and reduce thixotropic resistance within the moving tissue [21,22]. This allows the cyclist to maintain a higher power output at a lower metabolic cost, which explains the shift in the lactate curve without requiring changes in maximum oxygen uptake (VO_2_max). This is further supported by evidence suggesting that SMR after high-intensity training can significantly accelerate the clearance of lactate from the blood, a process vital for active recovery [27,80]. The compression generated by a foam roller may enhance local microcirculation and promote metabolic waste removal through altered interstitial pressure, facilitating the transport of byproducts out of the muscle-fascia unit. In our cohort, this likely lowered the metabolic cost of movement, allowing cyclists to maintain higher power outputs despite age-related declines in muscle-fascia compliance. The 76% larger effect observed at the anaerobic threshold further implies that SMR specifically optimizes high-intensity performance where tissue stiffness and thixotropic resistance play a dominant, limiting role.

This aligns with observations by Healey et al. [81], who found that post-training fatigue was significantly lower following foam rolling. Such a reduction in perceived fatigue may allow participants to increase acute training duration and intensity, potentially driving long-term performance gains. These results resonate with the findings of longitudinal studies which show that consistent SMR training enhances mechanical efficiency, specifically through improved torque effectiveness, leg strength symmetry, and pedal smoothness, even when metabolic markers like submaximal VO_2_ remain static [26,82]. In this context, a further analysis confirms that participants in the intervention group showed a significantly smaller increase in perceived training intensity and exertion compared to the control group, suggesting that SMR helps maintain mechanical output at a lower subjective cost [83].

However, the empirical landscape remains nuanced; for instance, Shalfawi et al. observed that while quadriceps myofascial release using mechanical roller-massagers showed potential benefits for blood lactate concentration and Wingate peak power in elite speed skaters, these indications did not translate into marked performance improvements, though notably, no negative effects were recorded [84]. Due to the lack of research investigating SMR specifically within longitudinal cycling interventions, evidence from other athletic disciplines must been considered. Studies involving varied training interventions, ranging from resistance training to diverse sports meta-analyses, consistently demonstrate the efficacy of SMR in trained populations [2,27,63,79,85,86]. This suggests that the physiological mechanisms of SMR are robust across different types of athletic stimuli.

Crucially, the reasons for these benefits may not be limited to one theoretical explanation, as the underlying mechanisms might be logically linked. For instance, an increase in the temperature of the tissue due to friction leads to a thixotropic response, which helps release adhesions. Simultaneously, increased blood flow may result in improved oxygenation, which may reduce the likelihood of trigger points being formed [1]. Moreover, by stimulating mechanoreceptors, SMR can shift the athlete’s state toward parasympathetic dominance, aiding in athlete recovery and potentially influencing the long-term adaptation to high training loads [21,22]. This reduction in systemic sympathetic drive may manifest as improved heart rate recovery following intense bouts. SMR post-exercise leads to a marked decrease in pain intensity and accelerates the restoration of muscle function, with the most pronounced benefits typically occurring 48 to 72 h after the training stimulus [2,86]. Practical experiments have shown that SMR can reduce soreness by up to 50% compared to untreated limbs, while simultaneously improving the range of motion (ROM) [78]. By mitigating the subjective feeling of exhaustion and muscle pain, athletes are empowered to return to high-intensity sessions sooner, facilitating a higher cumulative training load and better mechanical efficiency over time [82,83,87,88].

To summarize, these findings suggest that foam rolling does not show a uniform effect across endurance-related variables. Theoretically, the results indicate that the impact of SMR on the fascia and endurance-related outcomes are multidimensional. Based on our analysis, foam rolling does not appear to enhance cardiorespiratory capacity and efficiency directly, but it showed improvements for metabolic kinetics and mechanical performance. Supposedly, SMR acts as an indirect performance enhancer; while it may not provide an immediate “ergogenic boost” in power output, its ability to mitigate the negative side effects of high-intensity training, such as DOMS and metabolic fatigue, without impairing performance makes it a superior tool for the recovery phase compared to passive rest or static stretching [63,89]. In sum, our analysis reflects the broader inconsistencies in the empirical literature, as pointed out in [2,26] and emphasizes the need for further high-quality, well-controlled studies. 

### 4.1. Limitations

Although this study provides valuable insights into the long-term effects of SMR on endurance and performance, some limitations should be acknowledged. First, while descriptive statistics showed improvements in oxygen uptake, inferential statistics could not confirm these. These results must be interpreted within the context of the study’s statistical power of 72.4%, which falls slightly below the conventional 80% threshold. Therefore, the absence of significance in cardiorespiratory markers should be viewed as a potential Type II error (*β* = 27.6%) rather than a definitive lack of physiological effect. It must be emphasized that the smaller sample size (*n* = 32) is not just a formal limitation but significantly influences the interpretation of the results. Findings regarding VO_2_max and heart rate must therefore be interpreted with caution; they primarily serve to generate hypotheses for future, larger-scale studies (confirmatory trials) that can build upon the trends observed here.

A significant limitation that must be addressed is the baseline age imbalance between the experimental groups. The control group was, on average, approximately six years older than the intervention group (46.1 vs. 40.3 years). Since age is a known determinant of recovery rates, lactate kinetics [77], and overall training adaptation, this disparity represents a potential confounding factor that could theoretically bias results against the older control group. While we addressed this by including age as a covariate in our Linear Mixed-Effects Models (LMM) and found that the primary intervention effects on lactate slope and power output remained robust, the physiological influence of age on master-level athletes cannot be entirely dismissed. The slightly lower adaptive capacity of older muscle tissue might have exaggerated the relative success of the intervention group [73,76]. Future research should prioritize stricter age-matching during the randomization process to further isolate the effects of myofascial interventions.

Beyond these internal constraints, the generalizability of our findings must be discussed in the context of the broader scholarly literature. First, our results apply to trained recreational cyclists but may differ significantly for elite or professional athletes. In highly trained populations, some studies suggest a ‘ceiling effect’ for SMR; for instance, while we found significant gains in mechanical efficiency, studies on elite athletes often show that their recovery kinetics are already so optimized that additional myofascial interventions yield only marginal gains [2,26,84]. Second, the age-related context is vital. Our study focused on master-level cyclists, where we observed that SMR counteracts age-dependent fascial thickening [5]. In contrast, studies on younger active and healthy athletes (e.g., [19,20]) often report faster baseline recovery rates, potentially narrowing the ‘window of opportunity’ for SMR to show statistically significant long-term adaptations compared to our older cohort.

Furthermore, the exclusion of female athletes represents a critical gap. Research in sports medicine highlights that hormonal profiles, particularly estrogen, significantly influence collagen metabolism and fascial stiffness [6,21]. The efficacy of SMR likely follows gender-specific patterns that our data cannot reflect. Lastly, the specificity of the endurance discipline must be acknowledged. Unlike cycling, which is a non-weight-bearing activity, disciplines like running rely heavily on the stretch-shortening cycle (SSC) and the elastic energy storage of the fascia [87]. While we found improvements in mechanical power in cyclists, SMR research in runners suggests that excessive reduction in fascial stiffness could theoretically impact running economy differently than it does cycling power output [74,87,88].

Importantly, none of the underlying physiological mechanisms (e.g., direct blood flow measures, muscle stiffness via ultrasound, or EMG economy) were directly measured in this investigation. Therefore, the discussion of microcirculation, lactate clearance pathways, and fascial resistance remains speculative and serves as a plausible theoretical framework for the observed significant changes in lactate kinetics and power output.

Whereas SMR is traditionally associated with the reduction in muscle soreness (DOMS) and improvements in range of motion (ROM), these parameters were not the primary focus of this investigation as we focused on long-term physiological efficiency and metabolic adaptations during the first preparatory period (PP I). Our objective was to evaluate whether these tissue-level interventions translate into systemic output variables, such as lactate kinetics and mechanical power, which are more crucial for competitive road cycling performance. By utilizing a cohort of 32 male recreational cyclists, we aimed to evaluate these outcomes in a group where movement economy is already developed through regular training, ensuring the results reflect more than just a novelty stimulus. By prioritizing these performance-related outcomes, we aimed to bridge the gap between simple recovery techniques and long-term athletic development, even if this meant excluding traditional markers like perceived soreness of DOMS. However, the inclusion of ROM measurements could have provided additional information regarding the mechanical changes in the myofascial tissue and could have further supported our findings on mechanical efficiency.

Furthermore, a limitation common to many physical and behavioral interventions is the lack of participant blinding. The intervention group received an additional recovery routine, which may have induced expectancy or placebo effects. Such psychological factors can influence subjective parameters like the Rating of Perceived Exertion (RPE) or even physiological effort during performance testing. While the study primarily aimed to isolate physiological mechanisms through objective laboratory markers like lactate kinetics and spiroergometry, the perceived benefit of a proactive recovery strategy might have contributed to the observed results. Future research should consider implementing sham interventions for the control group to better differentiate between mechanical SMR effects and general expectancy effects.

Finally, our analysis could not settle the theoretical debate on the benefits of myofascial release techniques. The most compelling evidence of our study was shown in regard to metabolic kinetics, which aligns with physiological or mechanical explanations, while our results related to cardiorespiratory and cardiovascular variables such as maximal oxygen uptake or heart rate were inconclusive and could neither confirm nor rule out certain theoretical explanations. Consequently, future studies could put more emphasis on theoretical explanations and contribute to more theoretical clarity.

### 4.2. Practical Implications

Based on the findings of this six-month intervention, several practical recommendations for coaches, sports scientists, and competitive cyclists can be derived, moving beyond a “one-size-fits-all” approach to account for varying recovery rates and tissue characteristics. A primary application for the future is the development of tailored training programs where the strategic timing of SMR is adapted to the specific demands of the cycling discipline. For disciplines requiring high technical precision and explosive power, such as criterium racing or track cycling, the fascia roller could be utilized before training to “prime” the neuromuscular system, thereby improving torque effectiveness and ensuring a more fluid power delivery during high-cadence efforts. Conversely, in endurance-heavy disciplines like road racing or cross-country marathon, the focus should remain on post-exercise application. By utilizing SMR to influence the thixotropic state of the fascia and increase local perfusion, athletes can actively accelerate the clearance of metabolic byproducts, as evidenced by the significantly improved lactate kinetics observed in this study.

The evidence suggesting that SMR reduces subjective exertion and maintains mechanical integrity makes it an essential tool for multi-day stage races. Integrating approximately 15–20 min of targeted foam rolling into the daily post-race routine can help mitigate the cumulative “stiffening” effect often observed in the later stages of a race, thereby stabilizing mechanical power output over consecutive days. Beyond performance enhancement, the fascia roller serves as a highly accessible diagnostic instrument. Coaches can utilize regular SMR sessions to help athletes develop a “body map” of their muscle tension, allowing for the early identification of unilateral hotspots. This proactive approach to identifying leg strength imbalances and restricted fascial mobility is a cost-effective strategy to prevent common overuse injuries, such as iliotibial band (ITB) syndrome or patellar tendinopathy.

For optimal implementation, we recommend a high-volume, slow-speed protocol (approx. 35–40 min per session, rolling at 3 cm/s) using standardized video-guided instructions. This ensures that the 1:1 real-time speed and metabolic intensity targets are met consistently. Finally, since the positive effects on lactate and power were robust even when controlling for age, SMR represents a vital supplemental tool for master-level cyclists to maintain mechanical efficiency and long-term performance despite age-related physiological variations and the naturally declining rate of recovery associated with advancing age.

## 5. Conclusions

The present study suggests that the integration of SMR into the training regimen of road cyclists may not produce a uniform ergogenic effect. In summary, this pilot study provides initial evidence that SMR may influence efficiency (lactate kinetics, mechanical power), while no statistical significance was achieved for the central cardiorespiratory parameters in this exploratory setting. The lack of significant changes in systemic cardiorespiratory markers (VO_2_max, submaximal heart rate) indicates that localized myofascial interventions might not directly shift an athlete’s central cardiovascular “ceiling,” which remains primarily driven by aerobic conditioning.

However, it must be emphasized that the limited sample size (*n* = 32) and the resulting post hoc power of 72.4% influence the interpretation of these findings. The absence of significance in VO_2_max and heart rate should be viewed with caution and not as definitive evidence of a lack of effect, as the study may have been underpowered to detect moderate but meaningful changes (Type II error risk). Therefore, these results for central variables should be interpreted as exploratory trends.

Nevertheless, the significant improvements observed in metabolic kinetics (H3) and mechanical power output (H4) may point toward a shift in efficiency. While the findings are consistent with improved metabolic and mechanical efficiency, specific underlying pathways, ranging from directly enhanced microcirculation to reduced mechanical fascial resistance, remain partially speculative as these parameters were not directly measured. However, supported by recent evidence, it is hypothesized that the compression generated by foam rolling could enhance arterial perfusion and microcirculation, thereby potentially supporting an improved lactate clearance. By maintaining fascial fluidity and reducing thixotropic resistance within the lower-limb musculature, SMR might help optimize the mechanical efficiency of the pedal stroke. This could allow cyclists to translate their existing cardiorespiratory capacity more effectively into mechanical power output (watts) under specific metabolic conditions.

Despite these promising indications, the results reflect the broader scholarly inconsistencies regarding SMR. While SMR appears to facilitate an improved lactate metabolism and mechanical efficiency, direct cardiorespiratory advantages remain limited in this context. Certain limitations, such as the sample size (*n* = 16 per group), a male cohort, and the lack of long-term follow-up, must be considered when interpreting these findings.

Future research should aim for larger, confirmatory cohorts to increase statistical power and allow for even more robust modeling of physiological covariates (e.g., baseline flexibility or muscle stiffness), ensuring that training adaptations are not confounded by individual baseline differences or age-dependent physiological baselines. In sum, the value of SMR for cyclists lies not in an immediate performance boost but in its ability to facilitate metabolic recovery and preserve mechanical efficiency, making it an essential instrument for long-term performance maintenance.

## Figures and Tables

**Table 1:** Baseline characteristics and anthropometric data of the study participants (Mean ± *SD*). (Source: own illustration/Python).

Characteristic	Intervention Group (*n* = 16)	Control Group (*n* = 16)
Age (years)	40.3 ± 9.2	46.1 ± 8.4
Body Mass (kg)	78.4 ± 8.1	79.2 ± 7.6
BMI	24.12 ± 2.74	23.82 ± 1.63
VO_2_max (mL/min/kg)	46.7 ± 7.3	47.1 ± 6.8
Training Volume (h/week)	8.8 ± 1.1	9.1 ± 0.9
Weekly Mileage (km/week)	268.4 ± 34.2	275.6 ± 29.8
Annual Mileage (km/year)	13,420 ± 1710	13,780 ± 1490
Cycling Experience (years)	12.4 ± 6.5	13.8 ± 7.2

**Table 2:** Standardized Testing Protocol and Procedural Sequence (BL = Baseline test, P1 = Post-test 1, P2 = Post-test 2, Tr. = Training) (Source: own illustration/Python).

Sequence	Category	Parameter	Timepoint(s)	Key Protocol Detail
Block 1	Anthropometry	Body Height	BL	Measured to 0.1 cm (Seca 220).
	Body Mass	Weight & Distribution	BL, P1, P2	0.1 kg accuracy; Dual scales for L/R balance.
	Circumferences	Thigh & Calf	BL, P1, P2	Mid-thigh/calf widest point (bilateral).
	Tissue Analysis	L-BIA (Quadriceps)	BL, P1, P2	Supine; 5 cm above patella; R, Xc, Phase Angle.
Block 2	Performance	Spiroergometry	BL, P2	Incremental test (100 W + 50 W) every 3 min.
	Performance	Lactate Thresholds	BL, P2	Earlobe; Mader method (2 and 4 mmol/L).
	Mechanics	Power & Symmetry	BL, P2 (+Tr.)	Garmin^®^ Vector 2 (bilateral power output).
Block 3	Subjective	Pain & Overload	BL, P1, P2	Questionnaire on pain/overuse symptoms.
Monitoring	Load Tracking	Intensity & Load	Continuous	TRIMP model, 10-point Borg scale, NRS for Blackroll^®^, Training/Food diaries.

**Table 3:** Descriptive statistics of circumferences of the upper and lower legs and the left and right weight distribution for both groups at the baseline test and the post-test (Source: own illustration/Python).

		Baseline Test		Post-Test	
Variables	Statistics	Intervention	Control	Intervention	Control
BMI	Mean	24.12	23.82	24	24
	SD	2.74	1.63	2.6	1.3
Circumference lower leg left in cm	Mean	36.82	36.87	36.91	36.88
	SD	1.78	1.86	1.81	1.42
	Min.	34.5	34.7	34.5	34.9
	Max.	41.1	41.1	42	39
Circumference lower leg right in cm	Mean	37.23	37.39	37.04	37.08
	SD	2.10	2.56	1.92	1.87
	Min.	34.2	34.1	34.2	34.1
	Max.	41.5	42.1	42.1	40.0
Circumference upper leg left in cm	Mean	52.76	51.99	51.78	52.37
	SD	2.97	2.17	4.83	1.79
	Min.	48.3	48.7	36.9	49.0
	Max.	58.4	55.2	57.7	55.4
Circumference upper leg right in cm	Mean	52.68	52.02	52.77	52.64
	SD	2.82	2.11	2.67	1.78
	Min.	46.8	49.0	48.3	49.8
	Max.	56.4	55.5	57.2	55.3
Weight distribution left side in kg	Mean	39.42	37.28	39.07	36.97
	SD	4.46	3.82	3.74	3.2
	Min.	31.1	32.4	32.8	32.8
	Max.	45.9	45.1	46.3	43.8
Weight distribution right side in kg	Mean	39.68	35.91	39.15	36.39
	SD	5.42	4.55	3.85	4.47
	Min.	31.4	30.1	34	30.3
	Max.	48.9	45.3	46.2	46.4

**Table 4:** Linear Mixed-Effects Model results for the impact of foam rolling on maximal oxygen uptake (Source: own illustration/Python).

Category	Variable	Coefficient (*β*)	Std. Error	z-Value	*p*-Value	95% Conf. Interval
Fixed Effects	Intercept	62.450	5.820	10.730	<0.001	[51.045, 73.855]
	Time	1.438	0.834	1.723	0.085	[−0.198, 3.073]
	Group	−0.385	2.110	−0.182	0.855	[−4.521, 3.751]
	Age	−0.342	0.124	−2.758	0.006	[−0.585, −0.099]
	Group × Time	1.404	1.180	1.190	0.234	[−0.909, 3.717]
Variance	Random Intercept	36.025	5.608			
	Residual Variance	5.570	0.744			
Model Fit	Marginal *R*^2^	0.031				
	Conditional *R*^2^	0.870				

**Table 5:** Linear Mixed-Effects Model results for the impact of foam rolling on heart rate measured in heart beats per minute at the aerobic level or lactate threshold of 2 mmol/L (Source: own illustration/Python).

Category	Variable	Coefficient (*β*)	Std. Error	z-Value	*p*-Value	95% Conf. Interval
Fixed Effects	Intercept	138.45	12.15	11.39	<0.001	[114.63, 162.27]
	Time	0.875	1.721	0.508	0.611	[−2.498, 4.248]
	Group	1.063	3.702	0.287	0.774	[−6.193, 8.319]
	Age	−0.125	0.165	−0.752	0.452	[−0.448, 0.198]
	Group × Time	−0.375	2.436	−0.154	0.878	[−5.15; 4.40]
Variance	Random Intercept	86.120	14.250			
	Residual Variance	23.730	3.120			
Model Fit	Marginal *R*^2^	0.007				
	Conditional *R*^2^	0.789				

**Table 6:** Linear Mixed-Effects Model results for the impact of foam rolling on heart rate measured in heart beats per minute at the anaerobic level or lactate threshold of 4 mmol/L (Source: own illustration/Python).

Category	Variable	Coefficient (*β*)	Std. Error	z-Value	*p*-Value	95% Conf. Interval
Fixed Effects	Intercept	175.820	11.450	15.355	<0.001	[153.378, 198.262]
	Time	0.750	0.627	1.196	0.232	[−0.479, 1.979]
	Group	0.312	1.185	0.263	0.791	[−2.011, 2.635]
	Age	−0.118	0.169	−0.698	0.485	[−0.449, 0.213]
	Group × Time	−0.750	0.887	−0.845	0.398	[−2.489, 0.989]
Variance	Random Intercept	97.148	12.420			
	Residual Variance	3.150	0.548			
Model Fit	Marginal *R*^2^	0.004				
	Conditional *R*^2^	0.969				

**Table 7:** Linear Mixed-Effects Model results for the impact of foam rolling on the lactate curve from 100 to 250 watts with 50 watts increasing every three minutes (Source: own illustration/Python).

Category	Variable	Coefficient (*β*)	Std. Error	z-Value	*p*-Value	95% Conf. Interval
Fixed Effects	Intercept	0.024	0.005	4.800	<0.001	[0.014, 0.034]
	Time	0.001	0.002	0.500	0.644	[−0.003, 0.005]
	Time	0.001	0.002	0.500	0.644	[−0.003, 0.005]
	Age	0.0001	0.0002	0.370	0.712	[−0.0003, 0.0005]
	Group × Time	−0.007	0.002	−2.870	0.004	[−0.011, −0.002]
Variance	Random Intercept	0.000021	0.000004			
	Residual Variance	0.000005	0.000001			
Model Fit	Marginal *R*^2^	0.145				
	Conditional *R*^2^	0.812				

**Table 8:** Linear Mixed-Effects Model results for the impact of foam rolling on cycling performance measured in watts per kg at the aerobic level (2 mmol/L threshold) (Source: own illustration/Python).

Category	Variable	Coefficient (*β*)	Std. Error	z-Value	*p*-Value	95% Conf. Interval
Fixed Effects	Intercept	3.125	0.210	14.881	<0.001	[2.713, 3.537]
	Time	0.055	0.026	2.115	0.032	[0.004, 0.106]
	Group	−0.015	0.078	−0.192	0.849	[−0.168, 0.138]
	Age	−0.012	0.005	−2.400	0.024	[−0.022, −0.002]
	Group × Time	0.145	0.067	2.163	0.031	[0.014, 0.276]
Variance	Random Intercept	0.325	0.042			
	Residual Variance	0.018	0.003			
Model Fit	Marginal *R*^2^	0.065				
	Conditional *R*^2^	0.954				

**Table 9:** Linear Mixed-Effects Model results for the impact of foam rolling on cycling performance measured in watts per kg at the anaerobic level (4 mmol/L threshold) (Source: own illustration/Python).

Category	Variable	Coefficient (*β*)	Std. Error	z-Value	*p*-Value	95% Conf. Interval
Fixed Effects	Intercept	3.820	0.245	15.592	<0.001	[3.340, 4.300]
	Time	0.082	0.035	2.343	0.022	[0.013, 0.151]
	Group	−0.025	0.082	−0.305	0.760	[−0.186, 0.136]
	Age	−0.015	0.006	−2.500	0.012	[−0.027, −0.003]
	Group × Time	0.256	0.095	2.695	0.007	[0.070, 0.442]
Variance	Random Intercept	0.412	0.052			
	Residual Variance	0.024	0.004			
Model Fit	Marginal *R*^2^	0.115				
	Conditional *R*^2^	0.902				

## Data Availability

The data are not publicly available due to privacy or ethical restrictions.
